# The Safety and Effectiveness of Early, Progressive Weight Bearing and Implant Choice after Traumatic Lower Extremity Fracture: A Systematic Review

**DOI:** 10.3390/bioengineering9120750

**Published:** 2022-12-01

**Authors:** Daniel W. Flowers, Erin McCallister, Ricki Christopherson, Erin Ware

**Affiliations:** 1 Program in Physical Therapy, LSU Health Shreveport, 1501 Kings Hwy, Shreveport, LA 71103, USA; 2 Department of Physical and Occupational Therapy, Adult Inpatient Division, Duke University Hospital, 2301 Erwin Rd, Durham, NC 27710, USA; 3 Health Sciences Library, LSU Health Shreveport, 1501 Kings Hwy, Shreveport, LA 71103, USA

**Keywords:** mechanotransduction, orthopaedic, orthopedic, physical therapy, gait, bone healing

## Abstract

The goal of this systematic review was to examine existing evidence on the effectiveness of early, progressive weight bearing on patients after traumatic lower extremity fractures and relate these findings to device/implant choice. A search of the literature in PubMed/Medline, Embase, Web of Science, and the Cochrane Library was performed through January 2022. Randomized controlled trials and non-randomized, prospective longitudinal investigations of early, progressive weight bearing in skeletally mature adults after traumatic lower extremity fracture were included in the search, with 21 publications included in the final analysis. A summary of the loading progressions used in each study, along with the primary and additional outcomes, is provided. The progression of weight bearing was variable, dependent on fracture location and hardware fixation; however, overall outcomes were good with few complications. Most studies scored “high” on the bias tools and were predominately performed without physical therapist investigators. Few studies have investigated early, progressive weight bearing in patients after traumatic lower extremity fractures. The available clinical evidence provides variable progression guidelines. Relatively few complications and improved patient function were observed in this review. More research is needed from a rehabilitation perspective to obtain graded progression recommendations, informed by basic science concepts and tissue loading principles.

## 1. Introduction

Persons who have sustained lower extremity fractures, especially those with post-traumatic injuries, are greatly limited in their function and quality of life. The period of immobility is based on the length of time required for bone healing, typically no less than six to eight weeks, with signs of boney repair usually evidenced around four weeks. Methods of restoring and maintaining function are critical, especially when considering the detrimental effects of rest on injured tissues [1]. A gap exists between the basic science knowledge concerning the benefit of progressive loading of injured bone and clinical practice, mostly due to the absence of guidelines on the application of the theory of mechanotransduction [1]. This gap presents an opportunity to improve the rehabilitation process through staged and sequential loading for optimal outcomes.

The Physical Stress Theory has been utilized by therapists to apply these concepts and enumerates 12 “fundamental principles” within the context of clinical practice [2]. Forces at the fracture site are influenced by mechanical loading through the process of mechanotransduction, wherein cells respond to mechanical stimuli in the extracellular matrix via signal transduction across the cell membrane. The magnitude and direction of these forces acting at the fracture site influence fracture healing, with varying responses observed depending on the size of the fracture gap. Axial loading, which is observed in activities such as weight bearing, is more effective at influencing fracture healing than other types of force input [3]. Osteocytes themselves respond to these transduced signals, with osteoblasts recruited during higher loading. The gap in current knowledge continues to be how to appropriately dose mechanical stimuli (e.g., weight bearing) in the clinical environment to maximize recovery via the implementation of this knowledge of molecular biology [4].

Williamson et al. [5] demonstrated that immediate weight bearing after tibial plateau plate fixation showed no detrimental effects as compared with delayed weight-bearing precautions; however, progressive weight bearing was not investigated. These authors admitted there is “little consensus on the optimum weight-bearing status” for such injuries [5]. A second study by Weng et al. [6] on extra-articular tibial fractures fixated with intramedullary nailing compared a delayed weight-bearing group (non-weight bearing for 28 days) and an immediate weight bearing-group (partial weight bearing) with the latter having fewer incidents of non-union and a quicker healing time. Therefore, it remains to be seen whether progressive weight bearing-programs aimed at improving function and recovery after traumatic lower extremity fracture are beneficial for patients. A systematic review is uniquely suited to determine whether the current available evidence provides physical therapists with adequate guidelines on weight-bearing progression post fracture fixation in order to maximize patient outcomes and recovery. Paired with the basic science principles enumerated above, this knowledge could play an effective role in orthopaedic clinical practice.

The primary objective of this systematic review was to examine the effects of early, progressive weight-bearing training for patients after traumatic lower extremity fractures. A review of progressive weight-bearing programs, and their associated outcomes, would help guide clinicians in developing progressive weight-bearing protocols, when appropriate, given the consideration of fracture patterns and the implant/device used in fixation. A secondary aim of this investigation was to relate the primary outcomes to the clinical choice of implant/device in the fracture management of patients. Therefore, our research question was whether early, progressively dosed weight bearing is beneficial for patients after traumatic lower extremity fracture, and whether patterns can be identified according to implant choice during the fixation of the fracture.

## 2. Materials and Methods

This systematic review is reported according to the Preferred Reporting Items for Systematic Reviews (PRISMA) 2020 guidelines [7]. A protocol was written and submitted to Prospero on 11 December 2020. It was registered on 11 January 2021 under the number CRD42021225776. There was no external funding for this project.

### 2.1. Search Strategy

For this review, the population examined included skeletally mature adults who had sustained traumatic lower extremity fractures. The intervention/exposure was early, progressive weight bearing post injury. The primary outcomes included weight-bearing timeline/precautions, standardized outcome measure scores, non-union and/or mal-union rates, and healing time. Additional outcomes included bone density, assistance level with gait and functional tasks, falls, and infection rates.

A search of the literature for relevant data began in November 2020, with the final searches occurring November 13th through 16th. A librarian (EW) experienced in conducting research for reviews developed and conducted the searches (Supplemental File S1). The following databases and registers were searched: PubMed/Medline via the PubMed interface (National Library of Medicine), Embase (searching Medline as well) via Embase.com (Elsevier), Web of Science’s Science Citation Index Expanded (Clarivate), and the Cochrane Library (John Wiley and Sons), searching both the Cochrane Database of Systematic Reviews and the Cochrane Register of Controlled Trials (which in turn searched PubMed, Embase, the International Clinical Trials Registry Platform, ClinicalTrials.gov, and CINAHL for registrations for controlled trials). No limitations or filters were used on any of the searches.

### 2.2. Study Selection

After the citations were deduplicated using Systematic Review Accelerator (https://sr-accelerator.com/#/deduplicator, accessed on 20 November 2020 and 18 January 2022), they were uploaded to Rayyan (https://www.rayyan.ai, accessed on 20 November 2020 and 18 January 2022), where two authors (DF and RC) screened titles and abstracts to determine if they met the inclusion criteria, with a third author (EM) acting as a tiebreaker for disagreements. The same authors performed the same roles in the next step, which was to read the full text of the articles procured through the title and abstract screening.

Approximately fourteen months after the initial searches were completed, the same databases were searched again for any articles that had been published in the interim. One author (EW) searched the same databases using the previously formulated search strategies, again deduplicated in SR-Accelerator, and uploaded the citations to Rayyan. The same two authors (DF and RC) screened the new titles and abstracts to determine if they met the inclusion criteria, with another (EM) acting as a tiebreaker. The full-text review was then completed in the same manner as before.

Inclusion criteria for selected studies included randomized controlled trials and prospective longitudinal investigations. Only those investigating early, progressive weight bearing after traumatic lower extremity fractures were included. Additionally, included studies had to have at least one of the predetermined primary or secondary outcomes. These criteria were applied during the full text reviews by three of the authors (DF, RC, and EM).

### 2.3. Data Extraction and Quality Assessment

Once the study selection decisions were finalized, data extraction of the primary and additional outcomes from the included articles was performed by two authors (DF and RC). A third author (EM) provided a secondary review. Bias screening was performed by a single author (EM). The Cochrane Risk of Bias 2.0 (RoB 2.0) tool was used to assess randomized controlled trials (RCTs), while the Risk Of Bias In Non-randomized Studies of Interventions (ROBINS-I) was used to assess studies of non-randomized controlled trials (non-RCTs).

### 2.4. Summary of Findings and Results

Three of the authors (DF, EM, and RC) examined the included articles for the sought-after outcome variables. These findings are provided in Tables S1–S3 and are formatted and reported as appropriate for each measure. Due to the heterogeneity of study designs, intervention programs, and outcomes assessed, a meta-analysis was unable to be performed. The authors have provided a narrative summary of the results of the review.

## 3. Results

**Figure 1:**
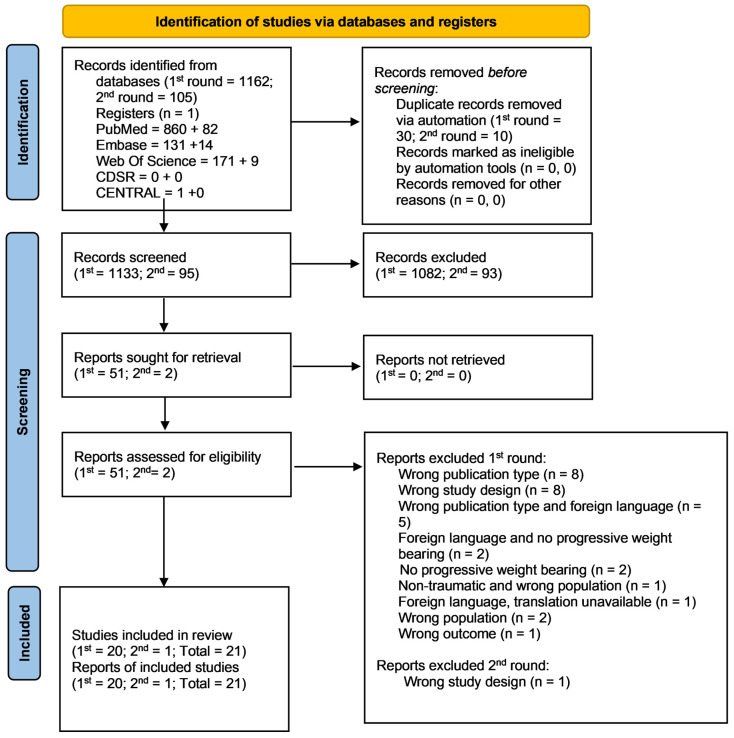
PRISMA Flowchart.

This review was performed from November 2020 to January 2022. A total of 1228 articles (after duplicate removal) were identified and screened. After screening and data collection, a total of 21 publications (eight RCTs and 13 non-RCTs) were included in the systematic review. Figure 1 presents the PRISMA flowchart for the screening and inclusion of the studies. The earliest date for any included study was 1980 [8], with the latest in 2021 [9], representing a publication range of 31 years. Fracture types investigated by the RCTs included three ankle, two calcaneal, and three tibial studies. Those from the non-RCTs included six femoral, five tibial (including two investigating tibial plateau fractures), and two calcaneal studies. There were no RCTs included investigating femoral fractures, no non-RCTs of the ankle, and no RCTs or non-RCTs examining midfoot or forefoot fractures. No meta-analysis was possible post review due to the limited number of articles (<10) per outcome measure sought in the review.

### 3.1. Quality Assessment

**Figure 2:**
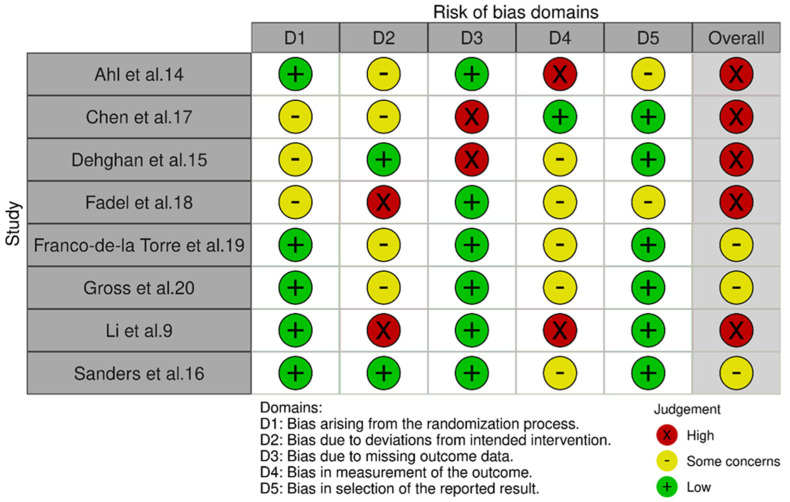
Randomized controlled trials risk of bias.

Figure 2 illustrates the bias of the randomized controlled studies assessed in this review [10], using the RoB2.0 tool. Four studies were deemed a “high” risk for bias, with the remaining three studies at “some concern” for bias. The leading cause of high risk for bias was missing outcome data, followed by bias in measurement outcome and deviation from intended intervention. All studies contained the information needed to complete the RoB2.0 bias assessment.

**Figure 3:**
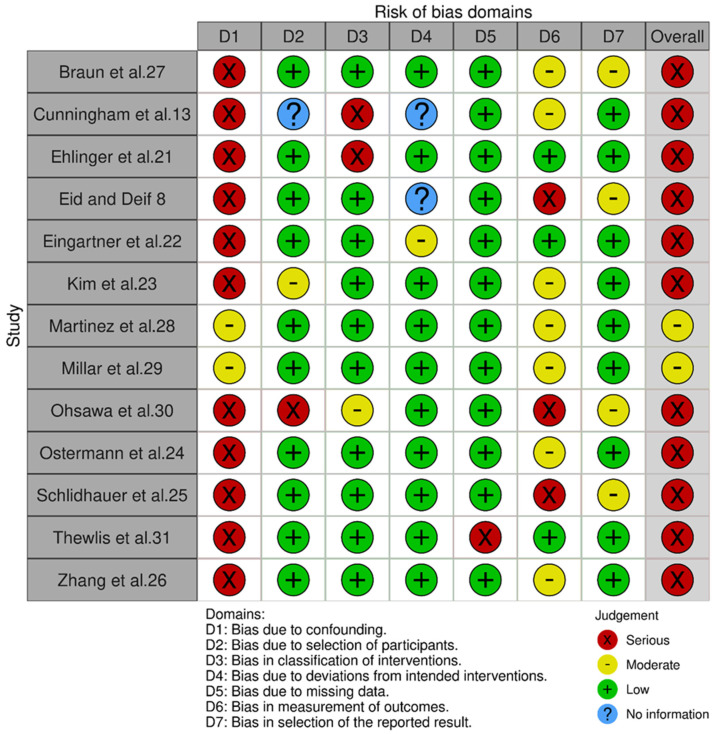
Non-randomized controlled trials risk of bias.

Figure 3 illustrates the bias of the non-randomized studies included in this review [10], using the ROBINS-I tool [11]. Eleven of the thirteen included studies were deemed to be at serious risk of bias, with the remaining two at moderate risk of bias. The most common domain to be at serious risk of bias was Domain 1: Bias Due to Confounding. Many studies failed to account for baseline prognostic factors such as BMI or comorbidities that could predict into which intervention group the participant was placed. Failure to measure or control for one important factor results in a rating of serious risk of bias for that study. According to the guiding document on using the ROBINS-I, a finding of “serious” risk in even one domain results in an overall risk of serious bias for that study [12]. The domains without any serious risk of bias were Domain 4 (bias due to deviations from intended interventions) and Domain 7 (bias in selection of the reported result). Two studies, Cunningham et al. [13] and Eid & Deif [8], were missing information required by the ROBINS-I, but both studies also had serious risk of bias in other domains and were therefore deemed to be at serious risk of bias.

### 3.2. Primary Outcomes

The primary outcomes included weight bearing-timeline/precautions, standardized outcome measure scores, non-union and/or mal-union rates, and healing times. Tables S2 and S3 provide study details and data on the primary outcomes for the RCTs and non-RCTs included in the analysis, respectively. Early, progressive weight bearing appears safe for those recovering from traumatic lower extremity fractures based on the low occurrence of complications across all studies.

Within the RCTs, the bone and joint involved appeared to influence the selection of weight-bearing progression. Early weight bearing for ankle fractures was successfully initiated immediately or up to two weeks post-operatively; these studies compared early weight bearing to delayed weight bearing beginning at weeks three through six [14,15,16]. All fractures allowed to bear weight early were fixed by open reduction internal fixation. All studies reported progression of weight bearing; however, no study detailed a controlled progression of weight-bearing status.

For calcaneal fractures, early weight bearing usually started at three to four weeks, while more traditional progression was started anywhere from six to 13 weeks [9,17]. Both studies initiated treatment with foot and ankle exercise and gradually progressed to full weight bearing by 12–13 weeks in the experimental groups. Chen et al. [17] used X-rays to determine when to initiate weight bearing in the delayed weight-bearing group, but did not describe similar procedures for the early weight-bearing group.

For tibial fractures, early weight bearing was started as soon as day one, with the delayed weight-bearing protocols initiating weight bearing at six weeks, or earlier, pending clinical examination [18,19,20]. With the earlier tibial weight-bearing progressions, there was some evidence that noise stimulation may be beneficial [19]. Only the study by Franco-de-la-Torre et al. [19] described a systematic approach to progressive weight bearing.

The progressive loading programs utilized in the non-RCT studies are harder to summarize due to a greater diversity in the methodology and application of the intervention. This is not surprising given the nature of the study designs, which ranged from case series to prospective longitudinal, single cohort investigations. Interestingly, many non-RCTs [8,21,22,23,24,25,26] provided more detail regarding standards for progressing weight bearing than the RCTs; this information was simply more varied within the same fracture type or location when compared with the RCTs.

The RCTs often compared early weight bearing as tolerated or partial weight bearing to delayed weight bearing. The non-RCTs utilized a variety of progressions. Few studies detailed weekly weight progression, and many RCTs [15,18,19,20] and non-RCTs [13,27,28,29,30,31] reported only early “weight bearing as tolerated” without further elaboration. These studies were included because the authors described limited weight bearing initially due to pain or assistive device use, but did not quantify the progression to full normal weight bearing, with the exception of Braun et al. [27] and Cunningham et al. [13] Further descriptions of the weight-bearing progressions can be found in Table S2 for RCTs and Table S3 for non-RCTs.

Healing time was difficult to judge in the included studies given that most authors defaulted to a generalized statement of when most/all fractures were healed. The lack of granular data for this primary outcome is most likely a result of data being collected only during routine post-fracture clinical visits in the orthopaedic surgery department. Unfortunately, this prevents any conclusions regarding the exact length of time needed for complete fracture healing. In general, when considering the RCT studies alone, successful, early weight-bearing progressions without negative outcomes were more likely in tibial fractures, followed by ankle and calcaneal, respectively.

There did not appear to be a standardized set of outcome measures used to track functional progress in people recovering from post-traumatic fractures. Other pathologies (e.g., hip and knee osteoarthritis) have recommended outcome measures for RCTs assessing participant performance-based outcomes [32,33]. There does not appear to be such a uniformity in RCTs for populations recovering from traumatic lower extremity fractures. One interesting finding was that the non-RCTs tended to use more outcome measures commonly utilized in rehabilitation practice (e.g., Knee Injury and Osteoarthritis Outcome Score, Timed Up and Go, Berg Balance Scale, etc.) while the RCTs used outcome measures more commonly used by physicians and surgeons (e.g., Olerud Molander Ankle Score, American Orthopedic Foot and Ankle Society Ankle–Hindfoot Scale, Short Musculoskeletal Functional Assessment, etc.).

### 3.3. Additional Outcomes

Additional outcomes included in this systematic review included bone density, assistance level with gait and functional tasks, falls, and infection rates. Table S1 details this data. Only three of the non-RCTs included data describing assistance levels with gait and functional tasks. Kim et al. [23] indicated crutches for post training for three patients due to peroneal nerve palsy; Braun et al. [27] indicated that 15 participants returned to their prior level of function by three months; and Eingartner et al. [22] reported that all participants were able to ambulate—three without assistive devices, eight with the use of a straight cane, and three with forearm crutches. Only two of the non-RCTs included data on falls, with Ehlinger et al. [21] reporting a single event and Eingartner et al. [22] reporting two.

Infection rate was the most reported secondary outcome: four of the RCTs [15,16,17,18] and three of the non-RCTs [21,22,25] reported an infection rate anywhere from zero to seven cases per study. Chen et al. [17] reported one infection per group, while Fadel et al. [18] had two in the plate osteosynthesis group and six in the Ilizarov fixation group. Sanders et al. [16] had six infections in the operative group. Dehghan et al. [15] were careful to point out that among the seven cases they reported, four were in the early weight-bearing groups and three in the late weight-bearing groups, with no statistically significant difference between the two. Regarding non-RCTs, Ehlinger et al. [21] and Eingartner et al. [22] reported two and zero infections, respectively. Schildhauer et al. [25], the only other non-RCT to report infection rates, noted four infections (11% of participants) adding that three participants were smokers.

### 3.4. Choice of Implant or Device

Four RCTs employed open reduction internal fixation (ORIF) management, with all four studies focused on foot/ankle fractures [9,14,15,16]. Three of the four studies [9,15,16] included data on outcome measures. Results indicated that those treated with ORIF and who were mobilized earlier had improved function on standardized outcome measures. Overall, there were low rates of non-union/mal-union [9,14,15,16]. Only Ahl et al. [14] reported healing time, with all healed at 18 months. One additional study examining distal tibial fractures, which compared Ilizarov external fixation with plate osteosynthesis, showed reduced functional outcomes, increased hardware failure, and increased healing time in the ORIF group [18]. For the non-RCTs, ORIF and compression plating showed good overall functional outcomes across all studies despite variability in the outcome measures utilized [23,25,29,31]. In the two non-RCTs that also reported on non-union/mal-union rates [23,25], no such rates were reported.

Only one RCT examining intramedullary nail (IMN) fixation reported on our primary outcomes [20], indicating no differences in functional outcomes or healing time compared with non-weight bearing. For non-union/mal-union, low rates of complication or no differences between groups were observed. The non-RCT study by Eingartner et al. [22] indicates fair scores on the Harris Hip Score were observed when IMN fixation is used in peri-prosthetic fractures. A higher rate of non-union in those ambulating earlier after femoral shaft fractures was observed [8], as opposed to only one delayed non-union after peri-prosthetic femur fractures [22].

The RCT by Fadel et al. [18] and the non-RCT by Zhang et al. [26] reported on outcomes, both indicating improved functional scores across their respective measures. The same was observed for percutaneous leverage with minimally invasive procedures described in the RCT by Chen et al. [17].

The only study reporting on cephalomedullary nail osteosynthesis did not report our primary outcomes [27]. Martinez et al. [28] and Ohsawa et al. [30] used fracture bracing and non-operative interventions, respectively. Finally, Eid and Deif [8] reported the use of open reduction preliminary fixation with Kirschner wires, with definitive fixation using cancellous screws, finding excellent scores on functional outcomes in those with femur fractures.

## 4. Discussion

The purpose of this systematic review was to determine whether early, progressively dosed weight bearing is beneficial for patients after traumatic lower extremity fracture. Our results indicate that little research has been done on the progressive dosing of weight bearing for this group of patients. Importantly, the literature that does utilize progressive early weight bearing provides little information that is helpful for enhancing the rehabilitation professional’s clinical decision-making.

Outcomes measured by healing time and complication rate indicate early, progressive weight bearing is safe in this population. However, few studies provided sufficient detail to replicate the weight-bearing dose or clinical decision-making used to progress weight bearing. Additionally, the lack of standardization in the studies regarding hardware fixation and fracture type makes the standardization of weight-bearing progressions difficult. All the included studies have either high/some concerns (RCTs) or moderate/serious (non-RCTs) overall bias ratings. The non-RCTs were unsurprisingly weak, with minimal control for confounding variables, while the RCTs were weaker in their intervention and outcomes methodology. This leads us to conclude that although the outcomes do indicate that early, progressive weight bearing does indicate good functional outcomes and low risk of complications, these findings are based on weak and sparse data.

One key finding of this review was that early, progressive weight bearing appears safe for patients following traumatic fracture. This finding is based on comparing the time to healing, mal-union or non-union rates, and secondary complications in delayed versus early weight bearing. However, our goal for this review was to elucidate not only the safety of early weight bearing but also a potential progressive dosing for weight bearing to assist rehabilitation clinicians in their clinical decision-making. As outlined in the Results section, there were few RCT or non-RCT studies that described either the exact weight-bearing progression, or the clinical standards used to advance weight-bearing status. Many studies used weight bearing “as tolerated” and allowed for offloading with a cast or assistive device to avoid pain during early weight bearing [13,15,18,19,20,27,28,29,30,31]. In addition, many older studies provided little detail on the rehabilitation aspect of the surgical intervention that accompanied the successful early progressive weight bearing. Therefore, the literature would benefit from RCTs that control for the weight-bearing dose, as well as RCTs that provide a more tissue-healing-based explanation for progressive weight bearing aside from progressing weight bearing “as tolerated” with pain as the only clinical benchmark. This research likely needs to be performed on multiple fracture types and locations, given the variety of findings in this review.

In addition, the studies included in this review used a wide variety of outcome measures. Within the RCTs, it appears that early weight-bearing protocols resulted in statistically significant improvements in function in the short term (three months post-operative fixation or post injury) but no significant improvements in longer-term follow-ups [15,20]. The non-RCT outcome measures provided more valuable outcome data for rehabilitation professionals because these studies used more rehabilitation-related outcome measures. However, the strength of evidence regarding improvement on outcome measures suffered from a lack of standardization and can be characterized as weak. Future studies would do well to include a variety of measures that characterize the patient’s body structure or function impairments, activity limitations, and participation limitations [34]. This variety would facilitate communication regarding patients’ functional ability between rehabilitation professionals and orthopaedic physicians.

The authors were unable to find conclusive patterns regarding the primary outcomes as related to implant and device type chosen for fixation, primarily due to the variability of facture types and fixation methods chosen. This finding, paired with the high degree of bias observed in many of the studies, makes recommendations on implant type for clinical use unwise. In short, providers should continue to focus on using appropriate implant and fixation methods as established by clinical norms and optimize functional recovery post-fracture fixation as dictated by what the load-sharing/load-bearing considerations indicate.

A concerning and unplanned finding of this review was the notable lack of participation of physical therapists, and the rehabilitation professions in general, in the planning, implementation, and publication of this clinical research topic. Of the 21 studies included, only three were published in rehabilitation-related journals [23,29,30], with one additional study published in a translational science journal [9]. All the remaining studies were published in orthopaedic, surgical, and/or medical journals. Only two articles had obvious [23] or suspected [30] authorship contributions from physical therapists or rehabilitation providers, although some articles did not provide author credentials. Only three of the included studies indicated a partnership with a rehabilitation department in their authorship affiliations beyond a cursory mention of rehabilitation services in the methodology from an otherwise physician-driven scholarly product. This area of research is so clinically applicable to the practice of orthopaedic physical therapy, and one that is ripe for translational and collaborative research opportunities to improve the clinical progression and resultant outcomes.

Although loading of bone post fracture has not been an area of research focus by physical therapists, there are those who have advocated for therapists to take leadership in the understanding of mechanotransduction. This could also help them appreciate the role they could potentially play in implementing optimally dosed mechanical stimuli in a clinical setting to improve recovery without causing injury [35,36]. Thompson et al. [35] describe the leadership and collaborative potential therapists could unlock in understanding these biological concepts, and specifically point out fractures as a potential area of practice worthy of applying these efforts. Tissue adaptation to mechanical loading is “underappreciated” [36], and could help explain the lack of therapists participating in such research as we have observed in this review. While research is still needed to answer how progressive loading may improve fracture healing and recovery, therapists have been active in a more basic science/histological approach to the effects of loading on bone health in clinical investigations [37,38].

This lack of research participation by physical therapists has not been observed when examining the literature surrounding loading of soft tissues post injury. On the contrary, when it comes to muscle, tendon, and ligamentous investigation, therapists have been counted among the leaders of impactful research on methodical, progressive loading of non-boney pathology. Physical therapists have been aggressively investigating soft tissue healing and providing practitioners with rehabilitation guidelines after lower extremity injury/surgical intervention. Examples include anterior cruciate ligament reconstruction graft type and loading considerations [39], anterior cruciate autograft harvesting with associated reconstruction [40], and controlled loading post-Achilles tendon rupture [41,42,43]. Their work on optimizing patient outcomes by progressing the research agenda related to soft tissue loading should be commended. Rehabilitation researchers in other fields of tissue healing could have a similar impact on the direction of rehabilitation research. Moritz and Ambrosio [44] noted a similar pattern to our own in the relative lack of representation of rehabilitation departments in the realm of regenerative medicine: only 0.6% of the National Institutes of Health-funded studies they accessed were sponsored by rehabilitation departments. Additionally, determining appropriate and optimal dosing, especially in the realm of regenerative medicine [45], continues to be an expansive landscape for translational and clinical research. Authors should discuss the results and how they can be interpreted from the perspective of previous studies and of the working hypotheses. The findings and their implications should be discussed in the broadest context possible. Future research directions may also be highlighted.

### 4.1. Implications for Clinical Practice

Physical therapists have a theoretical basis for progressing patients’ function following lower extremity fracture based upon general principles of tissue healing and basic science. Limited, low-level clinical research data are available to compliment this foundation. Otherwise, clinical research in this area is still in the nascent stage and requires continued investment and more active participation from researchers in our field. Therapists should maintain open dialogue with referring surgeons on weight-bearing progression and have a collaborative approach for safe and effective early, progressive weight bearing while conceding the lack of strong evidence for the practice in all clinical situations.

### 4.2. Study Limitations

There are several sources of limitations for this systematic review. First, regarding the findings of the review, the included studies report on weight-bearing progression for a variety of fracture types and surgical interventions. In addition, there is little detail on the actual dosing of the weight-bearing progression. This left the authors unable to draw useful conclusions on progressive weight bearing for clinicians based on fracture and/or surgical intervention. Second, many of the included studies have a high or serious risk for bias, even when taking their design (RCT or non-RCT) into account, whereby reducing the overall strength of the existing evidence, which is already limited. Finally, with relatively few studies having included physical therapists in the design, conduct, and publication of the results, the ability of the findings to be more applicable in therapists’ clinical practice is significantly limited.

## 5. Conclusions

Early progressive weight bearing for patients after certain traumatic lower extremity fractures may be safe with relatively few complications, especially when patients are followed closely by their surgeon throughout the weight-bearing progression. Most of the information available for the progression of these patients remains theoretical; the limited data available are from publications that score highly on methodological bias, resulting in inconsistent progression guidelines. The lack of progressive weight-bearing protocols in the literature means conclusive clinical recommendations are not possible at this point. Therefore, more research is warranted in this area, with partnerships between rehabilitation and surgical departments being of upmost importance.

## Data Availability

Not applicable.

## Supplementary Materials

The following supporting information can be downloaded at: https://www.mdpi.com/article/10.3390/bioengineering9120750/s1, Supplemental File S1: Search Strategy; Table S1: Outcomes from Included RCTs and Non-RCTs; Table S2: Study Characteristics and Primary Outcomes of Included RCTs; and Table S3: Study Characteristics and Primary Outcomes of Included Non-RCT Studies.
