# Supplementary material for: The Safety and Effectiveness of Early, Progressive Weight Bearing and Implant Choice after Traumatic Lower Extremity Fracture: A Systematic Review
Source: Bioengineering (Basel). 2022 Dec 1;9(12):750. doi: 10.3390/bioengineering9120750 (PMC9774827; doi:10.3390/bioengineering9120750)
Supplement: Supplementary file 1 [file bioengineering-09-00750-s001.zip › Supplemental File S1_Search Strategy.pdf]

PubMed Searched on November 13, 2020
(Pubmed searched via https://pubmed.ncbi.nlm.nih.gov/)

("progressive"[Title/Abstract] OR "progress"[Title/Abstract] OR "progression"[Title/Abstract] OR "progressively"[Title/Abstract] OR "progressing"[Title/Abstract] OR "continuous"[Title/Abstract] OR "continuously"[Title/Abstract] OR "increase"[Title/Abstract] OR "increasing"[Title/Abstract] OR "increased"[Title/Abstract] OR "growing"[Title/Abstract] OR "escalating"[Title/Abstract] OR "escalate"[Title/Abstract] OR "escalated"[Title/Abstract] OR "escalation"[Title/Abstract] OR "accelerating"[Title/Abstract] OR "accelerate"[Title/Abstract] OR "accelerated"[Title/Abstract] OR "acceleration"[Title/Abstract] OR "accelerations"[Title/Abstract] OR "rising"[Title/Abstract] OR "intesified"[Title/Abstract] OR ("intensification"[All Fields] OR "intensifications"[All Fields]) OR "intensifying"[Title/Abstract] OR "gradual"[Title/Abstract] OR "gradually"[Title/Abstract] OR "gradation"[Title/Abstract] OR "gradient"[Title/Abstract] OR "gradation"[Title/Abstract] OR "advancing"[Title/Abstract] OR "rising"[Title/Abstract] OR "mounting"[Title/Abstract] OR "graduated"[Title/Abstract] OR "titrated"[Title/Abstract] OR "titrate"[Title/Abstract] OR "titration"[Title/Abstract] OR ("increment"[All Fields] OR "incremented"[All Fields] OR "incrementing"[All Fields] OR "increments"[All Fields]) OR "increment"[Title/Abstract] OR "incremental"[Title/Abstract] OR "incrementally"[Title/Abstract]) AND ("weight bearing"[MeSH Terms] OR "weight bearing"[All Fields] OR "weight bearing"[All Fields] OR ("weight bearing"[MeSH Terms] OR "weight bearing"[All Fields] OR "weightbearing"[All Fields] OR "weightbear"[All Fields]) OR ("weight bearing"[MeSH Terms] OR "weight bearing"[All Fields] OR "loadbearing"[All Fields]) OR "load bearing"[All Fields] OR "load bearing"[All Fields] OR "Axial Loading"[All Fields] OR "Axial Loadings"[All Fields] OR (("weight s"[All Fields] OR "weighted"[All Fields] OR "weighting"[All Fields] OR "weightings"[All Fields] OR "weights and measures"[MeSH Terms] OR ("weights"[All Fields] AND "measures"[All Fields]) OR "weights and measures"[All Fields] OR "weight"[All Fields] OR "body weight"[MeSH Terms] OR ("body"[All Fields] AND "weight"[All Fields]) OR "body weight"[All Fields] OR "weights"[All Fields]) AND "bear*"[All Fields])) AND ("exercise"[MeSH Terms] OR "exercise"[All Fields] OR "exercises"[All Fields] OR "exercise therapy"[MeSH Terms] OR ("exercise"[All Fields] AND "therapy"[All Fields]) OR "exercise therapy"[All Fields] OR "exercise s"[All Fields] OR "exercised"[All Fields] OR "exerciser"[All Fields] OR "exercisers"[All Fields] OR "exercising"[All Fields] OR ("exercise"[MeSH Terms] OR "exercise"[All Fields] OR "exercises"[All Fields] OR "exercise therapy"[MeSH Terms] OR ("exercise"[All Fields] AND "therapy"[All Fields]) OR "exercise therapy"[All Fields] OR "exercise s"[All Fields] OR "exercised"[All Fields] OR "exerciser"[All Fields] OR "exercisers"[All Fields] OR "exercising"[All Fields]) OR ("exercise"[MeSH Terms] OR "exercise"[All Fields] OR "exercises"[All Fields] OR "exercise therapy"[MeSH Terms] OR ("exercise"[All Fields] AND "therapy"[All Fields]) OR "exercise therapy"[All Fields] OR "exercise s"[All Fields] OR "exercised"[All Fields] OR "exerciser"[All Fields] OR "exercisers"[All Fields] OR "exercising"[All Fields]) OR "excercised"[All Fields] OR ("clinical protocols"[MeSH Terms] OR ("clinical"[All Fields] AND "protocols"[All Fields]) OR "clinical protocols"[All Fields] OR "regimen"[All Fields] OR "regimen s"[All Fields]	860	860

OR "regimens"[All Fields]) OR (("physical examination"[MeSH Terms] OR ("physical"[All Fields] AND "examination"[All Fields]) OR "physical examination"[All Fields] OR "physical"[All Fields] OR "physically"[All Fields] OR "physicals"[All Fields]) AND ("therapeutics"[MeSH Terms] OR "therapeutics"[All Fields] OR "therapies"[All Fields] OR "therapy"[MeSH Subheading] OR "therapy"[All Fields] OR "therapy s"[All Fields] OR "therapys"[All Fields] OR ("medicin"[All Fields] OR "medicinal"[All Fields] OR "medicinally"[All Fields] OR "medicinals"[All Fields] OR "medicine"[MeSH Terms] OR "medicine"[All Fields] OR "medicine s"[All Fields] OR "medicines"[All Fields]) OR ("rehabilitation"[MeSH Terms] OR "rehabilitation"[All Fields] OR "rehab"[All Fields] OR "rehabs"[All Fields]) OR ("rehabilitant"[All Fields] OR "rehabilitants"[All Fields] OR "rehabilitate"[All Fields] OR "rehabilitated"[All Fields] OR "rehabilitates"[All Fields] OR "rehabilitating"[All Fields] OR "rehabilitation"[MeSH Terms] OR "rehabilitation"[All Fields] OR "rehabilitations"[All Fields] OR "rehabilitative"[All Fields] OR "rehabilitation"[MeSH Subheading] OR "rehabilitation s"[All Fields] OR "rehabilitational"[All Fields] OR "rehabilitator"[All Fields] OR "rehabilitators"[All Fields]))) OR ("physical therapy modalities"[MeSH Terms] OR ("physical"[All Fields] AND "therapy"[All Fields] AND "modalities"[All Fields]) OR "physical therapy modalities"[All Fields] OR "physiotherapies"[All Fields] OR "physiotherapy"[All Fields]) OR ("physical therapy modalities"[MeSH Terms] OR ("physical"[All Fields] AND "therapy"[All Fields] AND "modalities"[All Fields]) OR "physical therapy modalities"[All Fields] OR "physiotherapies"[All Fields] OR "physiotherapy"[All Fields]) OR ("therapeutical"[All Fields] OR "therapeutically"[All Fields] OR "therapeuticals"[All Fields] OR "therapeutics"[MeSH Terms] OR "therapeutics"[All Fields] OR "therapeutic"[All Fields]) OR ("therapeutics"[MeSH Terms] OR "therapeutics"[All Fields] OR "therapies"[All Fields] OR "therapy"[MeSH Subheading] OR "therapy"[All Fields] OR "therapy s"[All Fields] OR "therapys"[All Fields]) OR ("physiatric"[All Fields] OR "physical and rehabilitation medicine"[MeSH Terms] OR ("physical"[All Fields] AND "rehabilitation"[All Fields] AND "medicine"[All Fields]) OR "physical and rehabilitation medicine"[All Fields] OR "physiatrics"[All Fields]) OR ("physical and rehabilitation medicine"[MeSH Terms] OR ("physical"[All Fields] AND "rehabilitation"[All Fields] AND "medicine"[All Fields]) OR "physical and rehabilitation medicine"[All Fields] OR "physiatry"[All Fields])) AND (("fractur"[All Fields] OR "fractural"[All Fields] OR "fracture s"[All Fields] OR "fractures, bone"[MeSH Terms] OR ("fractures"[All Fields] AND "bone"[All Fields]) OR "bone fractures"[All Fields] OR "fracture"[All Fields] OR "fractured"[All Fields] OR "fractures"[All Fields] OR "fracturing"[All Fields] OR ("fractur"[All Fields] OR "fractural"[All Fields] OR "fracture s"[All Fields] OR "fractures, bone"[MeSH Terms] OR ("fractures"[All Fields] AND "bone"[All Fields]) OR "bone fractures"[All Fields] OR "fracture"[All Fields] OR "fractured"[All Fields] OR "fractures"[All Fields] OR "fracturing"[All Fields]) OR ("break"[All Fields] OR "breaking"[All Fields] OR "breakings"[All Fields] OR "breaks"[All Fields]) OR "broke"[All Fields] OR "broken"[All Fields] OR "fractures, bone"[MeSH Terms] OR "Fracture Healing"[MeSH Terms] OR "Fracture Healings"[All Fields] OR "healing fracture"[All Fields]) AND ("tibia"[MeSH Terms] OR "tibia"[All Fields] OR "tibias"[All Fields] OR "tibia s"[All Fields] OR "tibiae"[All Fields] OR ("fibula"[MeSH Terms] OR "fibula"[All Fields] OR "fibulas"[All Fields] OR "fibulae"[All Fields]) OR ("femur"[MeSH Terms] OR		

"femur"[All Fields] OR "femurs"[All Fields] OR "femur s"[All Fields] OR "femural"[All Fields] OR "femure"[All Fields]) OR ("patella"[MeSH Terms] OR "patella"[All Fields] OR "patellas"[All Fields] OR "patella s"[All Fields] OR "patellae"[All Fields]) OR ("talus"[MeSH Terms] OR "talus"[All Fields]) OR ("calcaneus"[MeSH Terms] OR "calcaneus"[All Fields]) OR ("cuboids"[All Fields] OR "tarsal bones"[MeSH Terms] OR ("tarsal"[All Fields] AND "bones"[All Fields]) OR "tarsal bones"[All Fields] OR "cuboid"[All Fields]) OR ("metatarsal bones"[MeSH Terms] OR ("metatarsal"[All Fields] AND "bones"[All Fields]) OR "metatarsal bones"[All Fields] OR "metatarsal"[All Fields] OR "metatarsals"[All Fields]) OR ("naviculare"[All Fields] OR "naviculars"[All Fields] OR "tarsal bones"[MeSH Terms] OR ("tarsal"[All Fields] AND "bones"[All Fields]) OR "tarsal bones"[All Fields] OR "navicular"[All Fields]) OR ("finger phalanges"[MeSH Terms] OR ("finger"[All Fields] AND "phalanges"[All Fields]) OR "finger phalanges"[All Fields] OR "phalanx"[All Fields] OR "phalanxes"[All Fields]) OR ("cuneiform"[All Fields] OR "cuneiforme"[All Fields] OR "cuneiforms"[All Fields]) OR ("ischium"[MeSH Terms] OR "ischium"[All Fields]) OR "tibiofibular"[All Fields]))		
("fractur"[All Fields] OR "fractural"[All Fields] OR "fracture s"[All Fields] OR "fractures, bone"[MeSH Terms] OR ("fractures"[All Fields] AND "bone"[All Fields]) OR "bone fractures"[All Fields] OR "fracture"[All Fields] OR "fractured"[All Fields] OR "fractures"[All Fields] OR "fracturing"[All Fields] OR ("fractur"[All Fields] OR "fractural"[All Fields] OR "fracture s"[All Fields] OR "fractures, bone"[MeSH Terms] OR ("fractures"[All Fields] AND "bone"[All Fields]) OR "bone fractures"[All Fields] OR "fracture"[All Fields] OR "fractured"[All Fields] OR "fractures"[All Fields] OR "fracturing"[All Fields]) OR ("break"[All Fields] OR "breaking"[All Fields] OR "breakings"[All Fields] OR "breaks"[All Fields]) OR "broke"[All Fields] OR "broken"[All Fields] OR "fractures, bone"[MeSH Terms] OR "Fracture Healing"[MeSH Terms] OR "Fracture Healings"[All Fields] OR "healing fracture"[All Fields]) AND ("tibia"[MeSH Terms] OR "tibia"[All Fields] OR "tibias"[All Fields] OR "tibia s"[All Fields] OR "tibiae"[All Fields] OR ("fibula"[MeSH Terms] OR "fibula"[All Fields] OR "fibulas"[All Fields] OR "fibulae"[All Fields]) OR ("femur"[MeSH Terms] OR "femur"[All Fields] OR "femurs"[All Fields] OR "femur s"[All Fields] OR "femural"[All Fields] OR "femure"[All Fields]) OR ("patella"[MeSH Terms] OR "patella"[All Fields] OR "patellas"[All Fields] OR "patella s"[All Fields] OR "patellae"[All Fields]) OR ("talus"[MeSH Terms] OR "talus"[All Fields]) OR ("calcaneus"[MeSH Terms] OR "calcaneus"[All Fields]) OR ("cuboids"[All Fields] OR "tarsal bones"[MeSH Terms] OR ("tarsal"[All Fields] AND "bones"[All Fields]) OR "tarsal bones"[All Fields] OR "cuboid"[All Fields]) OR ("metatarsal bones"[MeSH Terms] OR ("metatarsal"[All Fields] AND "bones"[All Fields]) OR "metatarsal bones"[All Fields] OR "metatarsal"[All Fields] OR "metatarsals"[All Fields]) OR ("naviculare"[All Fields] OR "naviculars"[All Fields] OR "tarsal bones"[MeSH Terms] OR ("tarsal"[All Fields] AND "bones"[All Fields]) OR "tarsal bones"[All Fields] OR "navicular"[All Fields]) OR ("finger phalanges"[MeSH Terms] OR ("finger"[All Fields] AND "phalanges"[All Fields]) OR "finger phalanges"[All Fields] OR "phalanx"[All Fields] OR "phalanxes"[All Fields]) OR ("cuneiform"[All Fields] OR "cuneiforme"[All Fields] OR "cuneiforms"[All Fields]) OR ("ischium"[MeSH Terms] OR "ischium"[All Fields]) OR "tibiofibular"[All Fields])	55324	
("progressive"[Title/Abstract] OR "progress"[Title/Abstract] OR "progression"[Title/Abstract] OR "progressively"[Title/Abstract] OR	12336	

"progressing"[Title/Abstract] OR "continuous"[Title/Abstract] OR "continuously"[Title/Abstract] OR "increase"[Title/Abstract] OR "increasing"[Title/Abstract] OR "increased"[Title/Abstract] OR "growing"[Title/Abstract] OR "escalating"[Title/Abstract] OR "escalate"[Title/Abstract] OR "escalated"[Title/Abstract] OR "escalation"[Title/Abstract] OR "accelerating"[Title/Abstract] OR "accelerate"[Title/Abstract] OR "accelerated"[Title/Abstract] OR "acceleration"[Title/Abstract] OR "accelerations"[Title/Abstract] OR "rising"[Title/Abstract] OR "intesified"[Title/Abstract] OR ("intensification"[All Fields] OR "intensifications"[All Fields]) OR "intensifying"[Title/Abstract] OR "gradual"[Title/Abstract] OR "gradually"[Title/Abstract] OR "gradation"[Title/Abstract] OR "gradient"[Title/Abstract] OR "gradation"[Title/Abstract] OR "advancing"[Title/Abstract] OR "rising"[Title/Abstract] OR "mounting"[Title/Abstract] OR "graduated"[Title/Abstract] OR "titrated"[Title/Abstract] OR "titrate"[Title/Abstract] OR "titration"[Title/Abstract] OR ("increment"[All Fields] OR "incremented"[All Fields] OR "incrementing"[All Fields] OR "increments"[All Fields]) OR "increment"[Title/Abstract] OR "incremental"[Title/Abstract] OR "incrementally"[Title/Abstract]) AND ("weight bearing"[MeSH Terms] OR "weight bearing"[All Fields] OR "weight bearing"[All Fields] OR ("weight bearing"[MeSH Terms] OR "weight bearing"[All Fields] OR "weightbearing"[All Fields] OR "weightbear"[All Fields]) OR ("weight bearing"[MeSH Terms] OR "weight bearing"[All Fields] OR "loadbearing"[All Fields]) OR "load bearing"[All Fields] OR "load bearing"[All Fields] OR "Axial Loading"[All Fields] OR "Axial Loadings"[All Fields] OR (("weight s"[All Fields] OR "weighted"[All Fields] OR "weighting"[All Fields] OR "weightings"[All Fields] OR "weights and measures"[MeSH Terms] OR ("weights"[All Fields] AND "measures"[All Fields]) OR "weights and measures"[All Fields] OR "weight"[All Fields] OR "body weight"[MeSH Terms] OR ("body"[All Fields] AND "weight"[All Fields]) OR "body weight"[All Fields] OR "weights"[All Fields]) AND "bear*"[All Fields])) AND ("exercise"[MeSH Terms] OR "exercise"[All Fields] OR "exercises"[All Fields] OR "exercise therapy"[MeSH Terms] OR ("exercise"[All Fields] AND "therapy"[All Fields]) OR "exercise therapy"[All Fields] OR "exercise s"[All Fields] OR "exercised"[All Fields] OR "exerciser"[All Fields] OR "exercisers"[All Fields] OR "exercising"[All Fields] OR ("exercise"[MeSH Terms] OR "exercise"[All Fields] OR "exercises"[All Fields] OR "exercise therapy"[MeSH Terms] OR ("exercise"[All Fields] AND "therapy"[All Fields]) OR "exercise therapy"[All Fields] OR "exercise s"[All Fields] OR "exercised"[All Fields] OR "exerciser"[All Fields] OR "exercisers"[All Fields] OR "exercising"[All Fields]) OR ("exercise"[MeSH Terms] OR "exercise"[All Fields] OR "exercises"[All Fields] OR "exercise therapy"[MeSH Terms] OR ("exercise"[All Fields] AND "therapy"[All Fields]) OR "exercise therapy"[All Fields] OR "exercise s"[All Fields] OR "exercised"[All Fields] OR "exerciser"[All Fields] OR "exercisers"[All Fields] OR "exercising"[All Fields]) OR "excercised"[All Fields] OR ("clinical protocols"[MeSH Terms] OR ("clinical"[All Fields] AND "protocols"[All Fields]) OR "clinical protocols"[All Fields] OR "regimen"[All Fields] OR "regimen s"[All Fields] OR "regimens"[All Fields]) OR (("physical examination"[MeSH Terms] OR ("physical"[All Fields] AND "examination"[All Fields]) OR "physical examination"[All Fields] OR "physical"[All Fields] OR "physically"[All Fields] OR "physicals"[All Fields]) AND ("therapeutics"[MeSH Terms] OR "therapeutics"[All Fields] OR "therapies"[All Fields] OR		

"therapy"[MeSH Subheading] OR "therapy"[All Fields] OR "therapy s"[All Fields] OR "therapys"[All Fields] OR ("medicin"[All Fields] OR "medicinal"[All Fields] OR "medicinally"[All Fields] OR "medicinals"[All Fields] OR "medicine"[MeSH Terms] OR "medicine"[All Fields] OR "medicine s"[All Fields] OR "medicines"[All Fields]) OR ("rehabilitation"[MeSH Terms] OR "rehabilitation"[All Fields] OR "rehab"[All Fields] OR "rehabs"[All Fields]) OR ("rehabilitant"[All Fields] OR "rehabilitants"[All Fields] OR "rehabilitate"[All Fields] OR "rehabilitated"[All Fields] OR "rehabilitates"[All Fields] OR "rehabilitating"[All Fields] OR "rehabilitation"[MeSH Terms] OR "rehabilitation"[All Fields] OR "rehabilitations"[All Fields] OR "rehabilitative"[All Fields] OR "rehabilitation"[MeSH Subheading] OR "rehabilitation s"[All Fields] OR "rehabilitational"[All Fields] OR "rehabilitator"[All Fields] OR "rehabilitators"[All Fields]))) OR ("physical therapy modalities"[MeSH Terms] OR ("physical"[All Fields] AND "therapy"[All Fields] AND "modalities"[All Fields]) OR "physical therapy modalities"[All Fields] OR "physiotherapies"[All Fields] OR "physiotherapy"[All Fields]) OR ("physical therapy modalities"[MeSH Terms] OR ("physical"[All Fields] AND "therapy"[All Fields] AND "modalities"[All Fields]) OR "physical therapy modalities"[All Fields] OR "physiotherapies"[All Fields] OR "physiotherapy"[All Fields]) OR ("therapeutical"[All Fields] OR "therapeutically"[All Fields] OR "therapeuticals"[All Fields] OR "therapeutics"[MeSH Terms] OR "therapeutics"[All Fields] OR "therapeutic"[All Fields]) OR ("therapeutics"[MeSH Terms] OR "therapeutics"[All Fields] OR "therapies"[All Fields] OR "therapy"[MeSH Subheading] OR "therapy"[All Fields] OR "therapy s"[All Fields] OR "therapys"[All Fields]) OR ("physiatric"[All Fields] OR "physical and rehabilitation medicine"[MeSH Terms] OR ("physical"[All Fields] AND "rehabilitation"[All Fields] AND "medicine"[All Fields]) OR "physical and rehabilitation medicine"[All Fields] OR "physiatrics"[All Fields]) OR ("physical and rehabilitation medicine"[MeSH Terms] OR ("physical"[All Fields] AND "rehabilitation"[All Fields] AND "medicine"[All Fields]) OR "physical and rehabilitation medicine"[All Fields] OR "physiatry"[All Fields]))		

Search was NOT limited to studies.
Search was NOT limited to English.

Embase Searched on November 16, 2020
(Embase searched Embase and Medline via Embase.com)

('fractur' OR 'fractural' OR 'fracture s' OR (('fractures'/exp OR 'fractures') AND ('bone'/exp OR 'bone')) OR 'bone fractures'/exp OR 'bone fractures' OR 'fracture'/exp OR 'fracture' OR 'fractured' OR 'fractures'/exp OR 'fractures' OR 'fracturing' OR 'break' OR 'breaking' OR 'breakings' OR 'breaks' OR 'broke' OR 'broken' OR 'fractures, bone'/exp OR 'fractures, bone' OR 'fracture healing'/exp OR 'fracture healing' OR 'fracture healings' OR 'healing fracture'/exp OR 'healing fracture') AND ('tibia'/exp OR 'tibia' OR 'tibias' OR 'tibia s' OR 'tibiae' OR 'fibula'/exp OR 'fibula' OR 'fibulas' OR 'fibulae' OR 'femur'/exp OR 'femur' OR 'femurs' OR 'femur s' OR 'femural' OR 'femure' OR 'patella'/exp OR 'patella' OR 'patellas' OR 'patella s' OR 'patellae' OR 'talus'/exp OR 'talus' OR 'calcaneus'/exp OR 'calcaneus' OR 'cuboids' OR 'cuboid' OR ('metatarsal' AND ('bones'/exp OR 'bones')) OR 'metatarsal bones'/exp OR 'metatarsal bones' OR 'metatarsal' OR	131	131

'metatarsals' OR 'naviculare' OR 'naviculars' OR ('tarsal' AND ('bones'/exp OR 'bones')) OR 'tarsal bones'/exp OR 'tarsal bones' OR 'navicular' OR (('finger'/exp OR 'finger') AND 'phalanges') OR 'finger phalanges'/exp OR 'finger phalanges' OR 'phalanx'/exp OR 'phalanx' OR 'phalanxes' OR 'cuneiform' OR 'cuneiforme' OR 'cuneiforms' OR 'ischium'/exp OR 'ischium' OR 'tibiofibular') AND (('progressive' OR 'progress' OR 'progression' OR 'progressively' OR 'progressing' OR 'continuous' OR 'continuously' OR 'increase' OR 'increasing' OR 'increased' OR 'growing' OR 'escalating' OR 'escalate' OR 'escalated' OR 'escalation' OR 'accelerating' OR 'accelerate' OR 'accelerated' OR 'acceleration' OR 'accelerations' OR 'intesified' OR 'intensification' OR 'intensifications' OR 'intensifying' OR 'gradual' OR 'gradually' OR 'gradient' OR 'gradation' OR 'advancing' OR 'rising' OR 'mounting' OR 'graduated' OR 'titrated' OR 'titrate' OR 'titration' OR 'incremented' OR 'incrementing' OR 'increments' OR 'increment' OR 'incremental' OR 'incrementally') NEAR/2 ('weight bearing' OR 'resistance training' OR 'axial loading'))		
('progressive' OR 'progress' OR 'progression' OR 'progressively' OR 'progressing' OR 'continuous' OR 'continuously' OR 'increase' OR 'increasing' OR 'increased' OR 'growing' OR 'escalating' OR 'escalate' OR 'escalated' OR 'escalation' OR 'accelerating' OR 'accelerate' OR 'accelerated' OR 'acceleration' OR 'accelerations' OR 'intesified' OR 'intensification' OR 'intensifications' OR 'intensifying' OR 'gradual' OR 'gradually' OR 'gradient' OR 'gradation' OR 'advancing' OR 'rising' OR 'mounting' OR 'graduated' OR 'titrated' OR 'titrate' OR 'titration' OR 'incremented' OR 'incrementing' OR 'increments' OR 'increment' OR 'incremental' OR 'incrementally') NEAR/2 ('weight bearing' OR 'resistance training' OR 'axial loading')	1696	
('fractur' OR 'fractural' OR 'fracture s' OR (('fractures'/exp OR 'fractures') AND ('bone'/exp OR 'bone')) OR 'bone fractures'/exp OR 'bone fractures' OR 'fracture'/exp OR 'fracture' OR 'fractured' OR 'fractures'/exp OR 'fractures' OR 'fracturing' OR 'break' OR 'breaking' OR 'breakings' OR 'breaks' OR 'broke' OR 'broken' OR 'fractures, bone'/exp OR 'fractures, bone' OR 'fracture healing'/exp OR 'fracture healing' OR 'fracture healings' OR 'healing fracture'/exp OR 'healing fracture') AND ('tibia'/exp OR 'tibia' OR 'tibias' OR 'tibia s' OR 'tibiae' OR 'fibula'/exp OR 'fibula' OR 'fibulas' OR 'fibulae' OR 'femur'/exp OR 'femur' OR 'femurs' OR 'femur s' OR 'femural' OR 'femure' OR 'patella'/exp OR 'patella' OR 'patellas' OR 'patella s' OR 'patellae' OR 'talus'/exp OR 'talus' OR 'calcaneus'/exp OR 'calcaneus' OR 'cuboids' OR 'cuboid' OR ('metatarsal' AND ('bones'/exp OR 'bones')) OR 'metatarsal bones'/exp OR 'metatarsal bones' OR 'metatarsal' OR 'metatarsals' OR 'naviculare' OR 'naviculars' OR ('tarsal' AND ('bones'/exp OR 'bones')) OR 'tarsal bones'/exp OR 'tarsal bones' OR 'navicular' OR (('finger'/exp OR 'finger') AND 'phalanges') OR 'finger phalanges'/exp OR 'finger phalanges' OR 'phalanx'/exp OR 'phalanx' OR 'phalanxes' OR 'cuneiform' OR 'cuneiforme' OR 'cuneiforms' OR 'ischium'/exp OR 'ischium' OR 'tibiofibular')	101476	

Search was NOT limited to studies.
Search was NOT limited to English.

Web of Science searched on November 16, 2020
(Web of Science searched Science Citation Index-Expanded)

TS= ("exercise" OR "exercise" OR "exercises" OR "exercise therapy" OR ("exercise" AND "therapy") OR "exercise therapy" OR "exercise s" OR "exercised" OR "exerciser" OR "exercisers" OR "exercising" OR ("exercise" OR "exercise" OR "exercises" OR "exercise therapy" OR ("exercise" AND "therapy") OR "exercise therapy" OR "exercise s" OR "exercised" OR "exerciser" OR "exercisers" OR "exercising") OR ("exercise" OR "exercise" OR "exercises" OR "exercise therapy" OR ("exercise" AND "therapy") OR "exercise therapy" OR "exercise s" OR "exercised" OR "exerciser" OR "exercisers" OR "exercising") OR "excercised" OR ("clinical protocols" OR ("clinical" AND "protocols") OR "clinical protocols" OR "regimen" OR "regimen s" OR "regimens") OR (("physical examination" OR ("physical" AND "examination") OR "physical examination" OR "physical" OR "physically" OR "physicals") AND ("therapeutics" OR "therapeutics" OR "therapies" OR "therapy" OR "therapy" OR "therapy s" OR "therapys" OR ("medicin" OR "medicinal" OR "medicinally" OR "medicinals" OR "medicine" OR "medicine" OR "medicine s" OR "medicines") OR ("rehabilitation" OR "rehabilitation" OR "rehab" OR "rehabs") OR ("rehabilitant" OR "rehabilitants" OR "rehabilitate" OR "rehabilitated" OR "rehabilitates" OR "rehabilitating" OR "rehabilitation" OR "rehabilitation" OR "rehabilitations" OR "rehabilitative" OR "rehabilitation" OR "rehabilitation s" OR "rehabilitational" OR "rehabilitator" OR "rehabilitators"))) OR ("physical therapy modalities" OR ("physical" AND "therapy" AND "modalities") OR "physical therapy modalities" OR "physiotherapies" OR "physiotherapy") OR ("physical therapy modalities" OR ("physical" AND "therapy" AND "modalities") OR "physical therapy modalities" OR "physiotherapies" OR "physiotherapy") OR ("therapeutical" OR "therapeutically" OR "therapeuticals" OR "therapeutics" OR "therapeutics" OR "therapeutic") OR ("therapeutics" OR "therapeutics" OR "therapies" OR "therapy" OR "therapy" OR "therapy s" OR "therapys") OR ("physiatric" OR "physical and rehabilitation medicine" OR ("physical" AND "rehabilitation" AND "medicine") OR "physical and rehabilitation medicine" OR "physiatrics") OR ("physical and rehabilitation medicine" OR ("physical" AND "rehabilitation" AND "medicine") OR "physical and rehabilitation medicine" OR "physiatry")) *Indexes=SCI-EXPANDED Timespan=All years*	171	171
TS= ("exercise" OR "exercise" OR "exercises" OR "exercise therapy" OR ("exercise" AND "therapy") OR "exercise therapy" OR "exercise s" OR "exercised" OR "exerciser" OR "exercisers" OR "exercising" OR ("exercise" OR "exercise" OR "exercises" OR "exercise therapy" OR ("exercise" AND "therapy") OR "exercise therapy" OR "exercise s" OR "exercised" OR "exerciser" OR "exercisers" OR "exercising") OR ("exercise" OR "exercise" OR "exercises"	3095497	

OR "exercise therapy" OR ("exercise" AND "therapy") OR "exercise therapy" OR "exercise s" OR "exercised" OR "exerciser" OR "exercisers" OR "exercising") OR "excercised" OR ("clinical protocols" OR ("clinical" AND "protocols") OR "clinical protocols" OR "regimen" OR "regimen s" OR "regimens") OR (("physical examination" OR ("physical" AND "examination") OR "physical examination" OR "physical" OR "physically" OR "physicals") AND ("therapeutics" OR "therapeutics" OR "therapies" OR "therapy" OR "therapy" OR "therapy s" OR "therapys" OR ("medicin" OR "medicinal" OR "medicinally" OR "medicinals" OR "medicine" OR "medicine" OR "medicine s" OR "medicines") OR ("rehabilitation" OR "rehabilitation" OR "rehab" OR "rehabs") OR ("rehabilitant" OR "rehabilitants" OR "rehabilitate" OR "rehabilitated" OR "rehabilitates" OR "rehabilitating" OR "rehabilitation" OR "rehabilitation" OR "rehabilitations" OR "rehabilitative" OR "rehabilitation" OR "rehabilitation s" OR "rehabilitational" OR "rehabilitator" OR "rehabilitators"))) OR ("physical therapy modalities" OR ("physical" AND "therapy" AND "modalities") OR "physical therapy modalities" OR "physiotherapies" OR "physiotherapy") OR ("physical therapy modalities" OR ("physical" AND "therapy" AND "modalities") OR "physical therapy modalities" OR "physiotherapies" OR "physiotherapy") OR ("therapeutical" OR "therapeutically" OR "therapeuticals" OR "therapeutics" OR "therapeutics" OR "therapeutic") OR ("therapeutics" OR "therapeutics" OR "therapies" OR "therapy" OR "therapy" OR "therapy s" OR "therapys") OR ("physiatric" OR "physical and rehabilitation medicine" OR ("physical" AND "rehabilitation" AND "medicine") OR "physical and rehabilitation medicine" OR "physiatrics") OR ("physical and rehabilitation medicine" OR ("physical" AND "rehabilitation" AND "medicine") OR "physical and rehabilitation medicine" OR "physiatry")) *Indexes=SCI-EXPANDED Timespan=All years*		
((fracture OR fractured OR fractures OR break OR broke OR broken) AND ("tibia" OR "tibia" OR "tibias" OR "tibia s" OR "tibiae" OR ("fibula" OR "fibula" OR "fibulas" OR "fibulae") OR ("femur" OR "femur" OR "femurs" OR "femur s" OR "femural" OR "femure") OR ("patella" OR "patella" OR "patellas" OR "patella s" OR "patellae") OR ("talus" OR "talus") OR ("calcaneus" OR "calcaneus") OR ("cuboids" OR "tarsal bones" OR ("tarsal" AND "bones") OR "tarsal bones" OR "cuboid") OR ("metatarsal bones" OR ("metatarsal" AND "bones") OR "metatarsal bones" OR "metatarsal" OR "metatarsals") OR ("naviculare" OR "naviculars" OR "tarsal bones" OR ("tarsal" AND "bones") OR "tarsal bones" OR "navicular") OR ("finger phalanges" OR ("finger" AND "phalanges") OR "finger phalanges" OR "phalanx" OR "phalanxes") OR ("cuneiform" OR "cuneiforme" OR "cuneiforms") OR ("ischium" OR "ischium") OR "tibiofibular")) AND (("progressive" OR "progress" OR "progression" OR "progressively" OR "progressing" OR "continuous" OR "continuously" OR "increase"	673	

OR "increasing" OR "increased" OR "growing" OR "escalating" OR "escalate" OR "escalated" OR "escalation" OR "accelerating" OR "accelerate" OR "accelerated" OR "acceleration" OR "accelerations" OR "rising" OR "intensified" OR ("intensification" OR "intensifications") OR "intensifying" OR "gradual" OR "gradually" OR "gradation" OR "gradient" OR "gradation" OR "advancing" OR "rising" OR "mounting" OR "graduated" OR "titrated" OR "titrate" OR "titration" OR ("increment" OR "incremented" OR "incrementing" OR "increments") OR "increment" OR "incremental" OR "incrementally") AND ("weight bearing" OR "weight bearing" OR "weight bearing" OR ("weight bearing" OR "weight bearing" OR "weightbearing" OR "weightbear") OR ("weight bearing" OR "weight bearing" OR "loadbearing") OR "load bearing" OR "load bearing" OR "Axial Loading" OR "Axial Loadings")) *Indexes=SCI-EXPANDED Timespan=All years*		

Search was NOT limited to studies.
Search was NOT limited to English.

Cochrane Reviews (Cochrane Database of Systematic Reviews) searched on November 16, 2020

("progressive weight bearing"):ti,ab,kw AND (fracture):ti,ab,kw AND (leg OR leg bone):ti,ab,kw	0	0
Leg OR leg bone	368	
fracture	233	
"progressive weight bearing"	0	

Search was NOT limited to studies.
Search was NOT limited to English only.

CENTRAL (Cochrane Central Register of Controlled Trials) searched on November 16, 2020 (CENTRAL searched PubMed, Embase, ICTRP, ClinicalTrials.gov, and CINAHL)

("progressive weight bearing"):ti,ab,kw AND (fracture):ti,ab,kw AND (leg OR leg bone):ti,ab,kw	1	1
Leg OR leg bone	24988	
fracture	24495	
"progressive weight bearing"	0	

WoS: *Timespan: 2020-11-17 to 2022-01-18 (Index Date)*
https://www.webofscience.com/wos/woscc/summary/885430e9-c8ab-4b63-b3a0-7ae876c3ce5f-1f7df9c6/date-descending/1

PubMed Searched on January 18, 2022
(Pubmed searched via https://pubmed.ncbi.nlm.nih.gov/) (PubMed: Results were manually searched because, when date limited, only 55 citations appeared. It should have been 82, and, in fact, when compared to the first search results, the 82 newest were not matched.)

("progressive"[Title/Abstract] OR "progress"[Title/Abstract] OR "progression"[Title/Abstract] OR "progressively"[Title/Abstract] OR "progressing"[Title/Abstract] OR "continuous"[Title/Abstract] OR	942	942

"continuously"[Title/Abstract] OR "increase"[Title/Abstract] OR "increasing"[Title/Abstract] OR "increased"[Title/Abstract] OR "growing"[Title/Abstract] OR "escalating"[Title/Abstract] OR "escalate"[Title/Abstract] OR "escalated"[Title/Abstract] OR "escalation"[Title/Abstract] OR "accelerating"[Title/Abstract] OR "accelerate"[Title/Abstract] OR "accelerated"[Title/Abstract] OR "acceleration"[Title/Abstract] OR "accelerations"[Title/Abstract] OR "rising"[Title/Abstract] OR "intesified"[Title/Abstract] OR ("intensification"[All Fields] OR "intensifications"[All Fields]) OR "intensifying"[Title/Abstract] OR "gradual"[Title/Abstract] OR "gradually"[Title/Abstract] OR "gradation"[Title/Abstract] OR "gradient"[Title/Abstract] OR "gradation"[Title/Abstract] OR "advancing"[Title/Abstract] OR "rising"[Title/Abstract] OR "mounting"[Title/Abstract] OR "graduated"[Title/Abstract] OR "titrated"[Title/Abstract] OR "titrate"[Title/Abstract] OR "titration"[Title/Abstract] OR ("increment"[All Fields] OR "incremented"[All Fields] OR "incrementing"[All Fields] OR "increments"[All Fields]) OR "increment"[Title/Abstract] OR "incremental"[Title/Abstract] OR "incrementally"[Title/Abstract]) AND ("weight bearing"[MeSH Terms] OR "weight bearing"[All Fields] OR "weight bearing"[All Fields] OR ("weight bearing"[MeSH Terms] OR "weight bearing"[All Fields] OR "weightbearing"[All Fields] OR "weightbear"[All Fields]) OR ("weight bearing"[MeSH Terms] OR "weight bearing"[All Fields] OR "loadbearing"[All Fields]) OR "load bearing"[All Fields] OR "load bearing"[All Fields] OR "Axial Loading"[All Fields] OR "Axial Loadings"[All Fields] OR (("weight s"[All Fields] OR "weighted"[All Fields] OR "weighting"[All Fields] OR "weightings"[All Fields] OR "weights and measures"[MeSH Terms] OR ("weights"[All Fields] AND "measures"[All Fields]) OR "weights and measures"[All Fields] OR "weight"[All Fields] OR "body weight"[MeSH Terms] OR ("body"[All Fields] AND "weight"[All Fields]) OR "body weight"[All Fields] OR "weights"[All Fields]) AND "bear*"[All Fields])) AND ("exercise"[MeSH Terms] OR "exercise"[All Fields] OR "exercises"[All Fields] OR "exercise therapy"[MeSH Terms] OR ("exercise"[All Fields] AND "therapy"[All Fields]) OR "exercise therapy"[All Fields] OR "exercise s"[All Fields] OR "exercised"[All Fields] OR "exerciser"[All Fields] OR "exercisers"[All Fields] OR "exercising"[All Fields] OR ("exercise"[MeSH Terms] OR "exercise"[All Fields] OR "exercises"[All Fields] OR "exercise therapy"[MeSH Terms] OR ("exercise"[All Fields] AND "therapy"[All Fields]) OR "exercise therapy"[All Fields] OR "exercise s"[All Fields] OR "exercised"[All Fields] OR "exerciser"[All Fields] OR "exercisers"[All Fields] OR "exercising"[All Fields]) OR ("exercise"[MeSH Terms] OR "exercise"[All Fields] OR "exercises"[All Fields] OR "exercise therapy"[MeSH Terms] OR ("exercise"[All Fields] AND "therapy"[All Fields]) OR "exercise therapy"[All Fields] OR "exercise s"[All Fields] OR "exercised"[All Fields] OR "exerciser"[All Fields] OR "exercisers"[All Fields] OR "exercising"[All Fields]) OR "excercised"[All Fields] OR ("clinical protocols"[MeSH Terms] OR ("clinical"[All Fields] AND "protocols"[All Fields]) OR "clinical protocols"[All Fields] OR "regimen"[All Fields] OR "regimen s"[All Fields] OR "regimens"[All Fields]) OR (("physical examination"[MeSH Terms] OR ("physical"[All Fields] AND "examination"[All Fields]) OR "physical examination"[All Fields] OR "physical"[All Fields] OR "physically"[All Fields] OR "physicals"[All Fields]) AND ("therapeutics"[MeSH Terms] OR "therapeutics"[All Fields] OR "therapies"[All Fields] OR "therapy"[MeSH Subheading] OR "therapy"[All Fields] OR "therapy		

s"[All Fields] OR "therapys"[All Fields] OR ("medicin"[All Fields] OR "medicinal"[All Fields] OR "medicinally"[All Fields] OR "medicinals"[All Fields] OR "medicine"[MeSH Terms] OR "medicine"[All Fields] OR "medicine s"[All Fields] OR "medicines"[All Fields]) OR ("rehabilitation"[MeSH Terms] OR "rehabilitation"[All Fields] OR "rehab"[All Fields] OR "rehabs"[All Fields]) OR ("rehabilitant"[All Fields] OR "rehabilitants"[All Fields] OR "rehabilitate"[All Fields] OR "rehabilitated"[All Fields] OR "rehabilitates"[All Fields] OR "rehabilitating"[All Fields] OR "rehabilitation"[MeSH Terms] OR "rehabilitation"[All Fields] OR "rehabilitations"[All Fields] OR "rehabilitative"[All Fields] OR "rehabilitation"[MeSH Subheading] OR "rehabilitation s"[All Fields] OR "rehabilitational"[All Fields] OR "rehabilitator"[All Fields] OR "rehabilitators"[All Fields]))) OR ("physical therapy modalities"[MeSH Terms] OR ("physical"[All Fields] AND "therapy"[All Fields] AND "modalities"[All Fields]) OR "physical therapy modalities"[All Fields] OR "physiotherapies"[All Fields] OR "physiotherapy"[All Fields]) OR ("physical therapy modalities"[MeSH Terms] OR ("physical"[All Fields] AND "therapy"[All Fields] AND "modalities"[All Fields]) OR "physical therapy modalities"[All Fields] OR "physiotherapies"[All Fields] OR "physiotherapy"[All Fields]) OR ("therapeutical"[All Fields] OR "therapeutically"[All Fields] OR "therapeuticals"[All Fields] OR "therapeutics"[MeSH Terms] OR "therapeutics"[All Fields] OR "therapeutic"[All Fields]) OR ("therapeutics"[MeSH Terms] OR "therapeutics"[All Fields] OR "therapies"[All Fields] OR "therapy"[MeSH Subheading] OR "therapy"[All Fields] OR "therapy s"[All Fields] OR "therapys"[All Fields]) OR ("physiatric"[All Fields] OR "physical and rehabilitation medicine"[MeSH Terms] OR ("physical"[All Fields] AND "rehabilitation"[All Fields] AND "medicine"[All Fields]) OR "physical and rehabilitation medicine"[All Fields] OR "physiatrics"[All Fields]) OR ("physical and rehabilitation medicine"[MeSH Terms] OR ("physical"[All Fields] AND "rehabilitation"[All Fields] AND "medicine"[All Fields]) OR "physical and rehabilitation medicine"[All Fields] OR "physiatry"[All Fields])) AND (("fractur"[All Fields] OR "fractural"[All Fields] OR "fracture s"[All Fields] OR "fractures, bone"[MeSH Terms] OR ("fractures"[All Fields] AND "bone"[All Fields]) OR "bone fractures"[All Fields] OR "fracture"[All Fields] OR "fractured"[All Fields] OR "fractures"[All Fields] OR "fracturing"[All Fields] OR ("fractur"[All Fields] OR "fractural"[All Fields] OR "fracture s"[All Fields] OR "fractures, bone"[MeSH Terms] OR ("fractures"[All Fields] AND "bone"[All Fields]) OR "bone fractures"[All Fields] OR "fracture"[All Fields] OR "fractured"[All Fields] OR "fractures"[All Fields] OR "fracturing"[All Fields]) OR ("break"[All Fields] OR "breaking"[All Fields] OR "breakings"[All Fields] OR "breaks"[All Fields]) OR "broke"[All Fields] OR "broken"[All Fields] OR "fractures, bone"[MeSH Terms] OR "Fracture Healing"[MeSH Terms] OR "Fracture Healings"[All Fields] OR "healing fracture"[All Fields]) AND ("tibia"[MeSH Terms] OR "tibia"[All Fields] OR "tibias"[All Fields] OR "tibia s"[All Fields] OR "tibiae"[All Fields] OR ("fibula"[MeSH Terms] OR "fibula"[All Fields] OR "fibulas"[All Fields] OR "fibulae"[All Fields]) OR ("femur"[MeSH Terms] OR "femur"[All Fields] OR "femurs"[All Fields] OR "femur s"[All Fields] OR "femural"[All Fields] OR "femure"[All Fields]) OR ("patella"[MeSH Terms] OR "patella"[All Fields] OR "patellas"[All Fields] OR "patella s"[All Fields] OR "patellae"[All Fields]) OR ("talus"[MeSH Terms] OR "talus"[All Fields]) OR ("calcaneus"[MeSH Terms] OR "calcaneus"[All Fields]) OR ("cuboids"[All Fields] OR "tarsal bones"[MeSH Terms] OR		

("tarsal"[All Fields] AND "bones"[All Fields]) OR "tarsal bones"[All Fields] OR "cuboid"[All Fields]) OR ("metatarsal bones"[MeSH Terms] OR ("metatarsal"[All Fields] AND "bones"[All Fields]) OR "metatarsal bones"[All Fields] OR "metatarsal"[All Fields] OR "metatarsals"[All Fields]) OR ("naviculare"[All Fields] OR "naviculars"[All Fields] OR "tarsal bones"[MeSH Terms] OR ("tarsal"[All Fields] AND "bones"[All Fields]) OR "tarsal bones"[All Fields] OR "navicular"[All Fields]) OR ("finger phalanges"[MeSH Terms] OR ("finger"[All Fields] AND "phalanges"[All Fields]) OR "finger phalanges"[All Fields] OR "phalanx"[All Fields] OR "phalanxes"[All Fields]) OR ("cuneiform"[All Fields] OR "cuneiforme"[All Fields] OR "cuneiforms"[All Fields]) OR ("ischium"[MeSH Terms] OR "ischium"[All Fields]) OR "tibiofibular"[All Fields]))		

Embase Searched on January 18, 2022
(Embase searched Embase, Medline, and Preprints via Embase.com)

('fractur' OR 'fractural' OR 'fracture s' OR (('fractures'/exp OR 'fractures') AND ('bone'/exp OR 'bone')) OR 'bone fractures'/exp OR 'bone fractures' OR 'fracture'/exp OR 'fracture' OR 'fractured' OR 'fractures'/exp OR 'fractures' OR 'fracturing' OR 'break' OR 'breaking' OR 'breakings' OR 'breaks' OR 'broke' OR 'broken' OR 'fractures, bone'/exp OR 'fractures, bone' OR 'fracture healing'/exp OR 'fracture healing' OR 'fracture healings' OR 'healing fracture'/exp OR 'healing fracture') AND ('tibia'/exp OR 'tibia' OR 'tibias' OR 'tibia s' OR 'tibiae' OR 'fibula'/exp OR 'fibula' OR 'fibulas' OR 'fibulae' OR 'femur'/exp OR 'femur' OR 'femurs' OR 'femur s' OR 'femural' OR 'femure' OR 'patella'/exp OR 'patella' OR 'patellas' OR 'patella s' OR 'patellae' OR 'talus'/exp OR 'talus' OR 'calcaneus'/exp OR 'calcaneus' OR 'cuboids' OR 'cuboid' OR ('metatarsal' AND ('bones'/exp OR 'bones')) OR 'metatarsal bones'/exp OR 'metatarsal bones' OR 'metatarsal' OR 'metatarsals' OR 'naviculare' OR 'naviculars' OR ('tarsal' AND ('bones'/exp OR 'bones')) OR 'tarsal bones'/exp OR 'tarsal bones' OR 'navicular' OR (('finger'/exp OR 'finger') AND 'phalanges') OR 'finger phalanges'/exp OR 'finger phalanges' OR 'phalanx'/exp OR 'phalanx' OR 'phalanxes' OR 'cuneiform' OR 'cuneiforme' OR 'cuneiforms' OR 'ischium'/exp OR 'ischium' OR 'tibiofibular') AND (('progressive' OR 'progress' OR 'progression' OR 'progressively' OR 'progressing' OR 'continuous' OR 'continuously' OR 'increase' OR 'increasing' OR 'increased' OR 'growing' OR 'escalating' OR 'escalate' OR 'escalated' OR 'escalation' OR 'accelerating' OR 'accelerate' OR 'accelerated' OR 'acceleration' OR 'accelerations' OR 'intesified' OR 'intensification' OR 'intensifications' OR 'intensifying' OR 'gradual' OR 'gradually' OR 'gradient' OR 'gradation' OR 'advancing' OR 'rising' OR 'mounting' OR 'graduated' OR 'titrated' OR 'titrate' OR 'titration' OR 'incremented' OR 'incrementing' OR 'increments' OR 'increment' OR 'incremental' OR 'incrementally') NEAR/2 ('weight bearing' OR 'resistance training' OR 'axial loading')) AND [17-11-2020]/sd NOT [19-01-2022]/sd	14	14
('fractur' OR 'fractural' OR 'fracture s' OR (('fractures'/exp OR 'fractures') AND ('bone'/exp OR 'bone')) OR 'bone fractures'/exp OR 'bone fractures' OR 'fracture'/exp OR 'fracture' OR 'fractured' OR 'fractures'/exp OR 'fractures' OR 'fracturing' OR 'break' OR 'breaking' OR 'breakings' OR 'breaks' OR 'broke' OR 'broken' OR 'fractures, bone'/exp OR 'fractures, bone' OR 'fracture healing'/exp OR 'fracture	141	

healing' OR 'fracture healings' OR 'healing fracture'/exp OR 'healing fracture') AND ('tibia'/exp OR 'tibia' OR 'tibias' OR 'tibia s' OR 'tibiae' OR 'fibula'/exp OR 'fibula' OR 'fibulas' OR 'fibulae' OR 'femur'/exp OR 'femur' OR 'femurs' OR 'femur s' OR 'femural' OR 'femure' OR 'patella'/exp OR 'patella' OR 'patellas' OR 'patella s' OR 'patellae' OR 'talus'/exp OR 'talus' OR 'calcaneus'/exp OR 'calcaneus' OR 'cuboids' OR 'cuboid' OR ('metatarsal' AND ('bones'/exp OR 'bones')) OR 'metatarsal bones'/exp OR 'metatarsal bones' OR 'metatarsal' OR 'metatarsals' OR 'naviculare' OR 'naviculars' OR ('tarsal' AND ('bones'/exp OR 'bones')) OR 'tarsal bones'/exp OR 'tarsal bones' OR 'navicular' OR (('finger'/exp OR 'finger') AND 'phalanges') OR 'finger phalanges'/exp OR 'finger phalanges' OR 'phalanx'/exp OR 'phalanx' OR 'phalanxes' OR 'cuneiform' OR 'cuneiforme' OR 'cuneiforms' OR 'ischium'/exp OR 'ischium' OR 'tibiofibular') AND (('progressive' OR 'progress' OR 'progression' OR 'progressively' OR 'progressing' OR 'continuous' OR 'continuously' OR 'increase' OR 'increasing' OR 'increased' OR 'growing' OR 'escalating' OR 'escalate' OR 'escalated' OR 'escalation' OR 'accelerating' OR 'accelerate' OR 'accelerated' OR 'acceleration' OR 'accelerations' OR 'intesified' OR 'intensification' OR 'intensifications' OR 'intensifying' OR 'gradual' OR 'gradually' OR 'gradient' OR 'gradation' OR 'advancing' OR 'rising' OR 'mounting' OR 'graduated' OR 'titrated' OR 'titrate' OR 'titration' OR 'incremented' OR 'incrementing' OR 'increments' OR 'increment' OR 'incremental' OR 'incrementally') NEAR/2 ('weight bearing' OR 'resistance training' OR 'axial loading'))		

Web of Science searched on January 18, 2022
(Web of Science searched Science Citation Index-Expanded)

TS= ("exercise" OR "exercise" OR "exercises" OR "exercise therapy" OR ("exercise" AND "therapy") OR "exercise therapy" OR "exercise s" OR "exercised" OR "exerciser" OR "exercisers" OR "exercising" OR ("exercise" OR "exercise" OR "exercises" OR "exercise therapy" OR ("exercise" AND "therapy") OR "exercise therapy" OR "exercise s" OR "exercised" OR "exerciser" OR "exercisers" OR "exercising") OR ("exercise" OR "exercise" OR "exercises" OR "exercise therapy" OR ("exercise" AND "therapy") OR "exercise therapy" OR "exercise s" OR "exercised" OR "exerciser" OR "exercisers" OR "exercising") OR "excercised" OR ("clinical protocols" OR ("clinical" AND "protocols") OR "clinical protocols" OR "regimen" OR "regimen s" OR "regimens") OR (("physical examination" OR ("physical" AND "examination") OR "physical examination" OR "physical" OR "physically" OR "physicals") AND ("therapeutics" OR "therapeutics" OR "therapies" OR "therapy" OR "therapy" OR "therapy s" OR "therapys" OR ("medicin" OR "medicinal" OR "medicinally" OR "medicinals" OR "medicine" OR "medicine" OR "medicine s" OR "medicines") OR ("rehabilitation" OR "rehabilitation" OR "rehab" OR "rehabs") OR ("rehabilitant" OR "rehabilitants" OR "rehabilitate"	9	9

OR "rehabilitated" OR "rehabilitates" OR "rehabilitating" OR "rehabilitation" OR "rehabilitation" OR "rehabilitations" OR "rehabilitative" OR "rehabilitation" OR "rehabilitation s" OR "rehabilitational" OR "rehabilitator" OR "rehabilitators"))) OR ("physical therapy modalities" OR ("physical" AND "therapy" AND "modalities") OR "physical therapy modalities" OR "physiotherapies" OR "physiotherapy") OR ("physical therapy modalities" OR ("physical" AND "therapy" AND "modalities") OR "physical therapy modalities" OR "physiotherapies" OR "physiotherapy") OR ("therapeutical" OR "therapeutically" OR "therapeuticals" OR "therapeutics" OR "therapeutics" OR "therapeutic") OR ("therapeutics" OR "therapeutics" OR "therapies" OR "therapy" OR "therapy" OR "therapy s" OR "therapys") OR ("physiatric" OR "physical and rehabilitation medicine" OR ("physical" AND "rehabilitation" AND "medicine") OR "physical and rehabilitation medicine" OR "physiatrics") OR ("physical and rehabilitation medicine" OR ("physical" AND "rehabilitation" AND "medicine") OR "physical and rehabilitation medicine" OR "physiatry")) *Indexes=SCI-EXPANDED* *Timespan: 2020-11-17 to 2022-01-18 (Index Date)*		
TS= ("exercise" OR "exercise" OR "exercises" OR "exercise therapy" OR ("exercise" AND "therapy") OR "exercise therapy" OR "exercise s" OR "exercised" OR "exerciser" OR "exercisers" OR "exercising" OR ("exercise" OR "exercise" OR "exercises" OR "exercise therapy" OR ("exercise" AND "therapy") OR "exercise therapy" OR "exercise s" OR "exercised" OR "exerciser" OR "exercisers" OR "exercising") OR ("exercise" OR "exercise" OR "exercises" OR "exercise therapy" OR ("exercise" AND "therapy") OR "exercise therapy" OR "exercise s" OR "exercised" OR "exerciser" OR "exercisers" OR "exercising") OR "excercised" OR ("clinical protocols" OR ("clinical" AND "protocols") OR "clinical protocols" OR "regimen" OR "regimen s" OR "regimens") OR (("physical examination" OR ("physical" AND "examination") OR "physical examination" OR "physical" OR "physically" OR "physicals") AND ("therapeutics" OR "therapeutics" OR "therapies" OR "therapy" OR "therapy" OR "therapy s" OR "therapys" OR ("medicin" OR "medicinal" OR "medicinally" OR "medicinals" OR "medicine" OR "medicine" OR "medicine s" OR "medicines") OR ("rehabilitation" OR "rehabilitation" OR "rehab" OR "rehabs") OR ("rehabilitant" OR "rehabilitants" OR "rehabilitate" OR "rehabilitated" OR "rehabilitates" OR "rehabilitating" OR "rehabilitation" OR "rehabilitation" OR "rehabilitations" OR "rehabilitative" OR "rehabilitation" OR "rehabilitation s" OR "rehabilitational" OR "rehabilitator" OR "rehabilitators"))) OR ("physical therapy modalities" OR ("physical" AND "therapy" AND "modalities") OR "physical therapy modalities" OR "physiotherapies" OR "physiotherapy") OR ("physical therapy modalities" OR ("physical" AND "therapy" AND "modalities") OR "physical therapy modalities" OR "physiotherapies" OR "physiotherapy") OR ("therapeutical" OR "therapeutically" OR	181	

"therapeuticals" OR "therapeutics" OR "therapeutics" OR "therapeutic") OR ("therapeutics" OR "therapeutics" OR "therapies" OR "therapy" OR "therapy" OR "therapy s" OR "therapys") OR ("physiatric" OR "physical and rehabilitation medicine" OR ("physical" AND "rehabilitation" AND "medicine") OR "physical and rehabilitation medicine" OR "physiatrics") OR ("physical and rehabilitation medicine" OR ("physical" AND "rehabilitation" AND "medicine") OR "physical and rehabilitation medicine" OR "physiatry")) *Indexes=SCI-EXPANDED Timespan=All years*		
