# Supplementary material for: The Safety and Effectiveness of Early, Progressive Weight Bearing and Implant Choice after Traumatic Lower Extremity Fracture: A Systematic Review
Source: Bioengineering (Basel). 2022 Dec 1;9(12):750. doi: 10.3390/bioengineering9120750 (PMC9774827; doi:10.3390/bioengineering9120750)
Supplement: Supplementary file 1 [file bioengineering-09-00750-s001.zip › Table S1_Outcomes from Included RCTs and Non-RCTs.pdf]

Table S1. Outcomes from Included RCTs and Non-RCTs[a]

Outcomes	Study Types	Study Sample Size, N	Fracture Location per Study	Outcome Specifics (RCT/non-RCT)
Standardized Outcome Measures	6 RCTs[9,15-18,20]/8 Non-RCTs[22-26,29-31]	Range: 3-110	2 Ankle<br>3 Femoral<br>4 Calcaneal<br>5 Tibial	Olerud Molander Ankle Score (2/0)[15,16]<br>Mazur Ankle Score (1/0)[18]<br>Kerr Calcaneal Score (0/1)[25]<br>Maryland Foot Function (1/0)[9]<br>American Orthopedic Foot and Ankle Society Ankle-Hindfoot Scale (2/0)[9,17]<br>Harris Hip Score (0/1)[22]<br>Knee Injury and Osteoarthritis Outcome Score (0/1)[29]<br>Short Musculoskeletal Function Assessment (1/0)[20]<br>Gait Speed (0/3)[23,29,31]<br>Timed-Up-And-Go (0/1)[23]<br>Berg Balance (0/1)[23]<br>Ambulation Functional Independence Measure (0/1)[30]<br>Neer Score (0/1)[24]<br>Unspecified Functional Score (0/1)[26]<br>Short Form 36 (2/0)[15,16]
Non-union and/or malunion rates	6 RCTs[9,14-16,18,20]/5 Non-RCTs[8,22,23,25,28]	Range: 3-110	3 Ankle<br>2 Femoral<br>2 Calcaneal<br>4 Tibial	Authors provided varying levels of specificity on this outcome, with some reporting group differences and statistical comparisons and others noting no loss of fixation. Early WBing groups fared better[16,18] or similar[9,15,20] on this outcome in direct comparisons with late WBing groups.
Healing time	3 RCTs[14,18,20]/1 Non-RCT[28]	Range: 40-108	1 Ankle<br>3 Tibial	Only one study[18] noted a difference in healing time between early and late WBing groups, with the early WBing group healing on average 66.5 days earlier.
Bone density	0	--	--	None of the included studies (RCTs or Non-RCTs) included bone density as an outcome measure.

Assistance level with gait and functional tasks	0 RCTs/3 Non-RCTs[22,23,27]	Range: 3-22	2 Femoral<br>1 Tibial	This outcome was more observational than standardized across studies. Most authors noted the use of assistive devices[22,23] and time to return to function.[27]
Falls	0 RCTs/2 Non-RCTs[21,22]	Range: 17-21	2 Femoral	Both studies that included this outcome investigated periprosthetic femoral fractures. A single fall was reported by Ehlinger et al.[21] and two by Eingartner et al.[22]
Infection Rates	4 RCTs[15-18]/3 Non-RCTs[21,22,25]	Range: 17-110	3 Ankle<br>2 Femoral<br>1 Calcaneal<br>1 Tibial	Infection rate was only counted as "reported" if authors stated a specific number of infections or noted none were observed. Infection rates ranged from 0-7 per study. In the RCTs, the early WBing groups had fewer or equal/not significantly different number of infections as the late WBing groups,[15,17] except in the study by Fadel et al.[18] and the one by Sanders et al.[16] where the early WBing group was the only one to include those post-operative.

[a] RCTs = randomized controlled trials; Non-RCTs = non-randomized controlled trials; WBing = weight bearing.
