# Supplementary material for: The Safety and Effectiveness of Early, Progressive Weight Bearing and Implant Choice after Traumatic Lower Extremity Fracture: A Systematic Review
Source: Bioengineering (Basel). 2022 Dec 1;9(12):750. doi: 10.3390/bioengineering9120750 (PMC9774827; doi:10.3390/bioengineering9120750)
Supplement: Supplementary file 1 [file bioengineering-09-00750-s001.zip › Table S2_Study Characteristics and Primary Outcomes of Included RCTs.pdf]

Table S2. Study Characteristics and Primary Outcomes of Included RCTs[a]

Study<br>Country<br>(Bone or joint)	Participants	N	Weight bearing timeline/precautions	Standardized outcome measure scores	Non-union/mal-union rates	Healing time
Ahl et al.<br>Sweden<br>(ankle fractures)	Injury Description:<br>46 dislocated lateral malleolus fractures; 53 bimalleolar fractures; all with ATFL rupture.<br>Fixation:<br>ORIF<br>Age: 55 years (19-81) F, 45 years (17-86) M	99<br>(61F, 38M)	Early versus late WBing groups.<br>Early: Started post-operative day one (N = 49).<br>Late: 4th-5th week post-operative (N = 50). Both using a below knee cast.	--	15/99 displacement of malleoli, 10 of which were minimal (<1mm). Fracture re-dislocation in three pts (2 in late WBing group; does not specify the third but was a trauma).	All healed at 18 months.
Chen et al.<br>China<br>(calcaneal fractures)	Injury Description:<br>Unilateral displaced intra-articular calcaneal fractures<br>Fixation:<br>Percutaneous leverage and minimally invasive fixation<br>Age: 40.3 years (study group) and 38.7 years (control group)	72	Study group: NWB post-operative days 0-3, day 3 rolling bottle under foot, WBing at week 3 (5 kg x at least 30 min), progressed gradually, full WBing at 12 weeks.<br>Control group: Partial WBing week six, full WBing after x-rays usually 12 weeks post-operative.	American Orthopedic Foot and Ankle Society (AOFAS) Ankle-Hindfoot Scale scores (assessed at post-op 12 months; 88.7+/-7.6 study group, 81.4+/-11.5 control group (t = 2.920, p = .003).	--	--

Dehghan et al<br>Canada<br>(ankle fractures)	Injury Description:<br>Unstable ankle fractures (lateral malleolus, medial malleolus, bimalleolar, trimalleolar)<br>Fixation:<br>ORIF<br>Age: 41.9 years	110<br>(51F, 59M)	N = 54 Late WBing Group, 56 Early WBing Group.<br>Late WBing Group: NWB, immobilization in below knee fiberglass cast at 2 weeks. WBing as tolerated at 6 weeks in boot orthosis weaned over 2-4 weeks.<br>Early WBing Group: WBing as tolerated at 2 weeks post op in boot orthosis, ankle range of motion 4 times/day while weaning boot at 6 weeks over 2-4 weeks.	Time to return to work: 2 weeks post operative, Late WBing Group = 33%, Early WBing Group = 16% (p=.05); No difference at any other point in return to work (48% at 6 weeks, 80% at 3 mons, 94% at 6 mons, 98% at 12 mons). Ankle Functional Outcome (Olerud/Molander ankle scores): Late WBing Group 32, Early WBing Group 45 (p=.0007) at 6 weeks; no statistical difference at subsequent visits and 1 year all achieved similar mean outcomes. Health Outcome SF-36: Physical Late WBing Group 42, Early WBing Group 51 (p=.0008), Mental Late WBing Group 54, Early WBing Group 66 (p=.0008), no difference at 3 months, 6 months Function Early WBing Group 79, Late WBing Group 72 (p=.07), Mental at 6 months Early WBing Group 84, Late WBing Group 79 (p=.08); 12 months Function Early WBing Group 85, Late WBing Group 79 (p=.04), Mental Early WBing Group 87, Late WBing Group 83 (p=.09).	No loss of fixation or reduction in either group.	--
Fadel et al<br>Egypt<br>(distal tibia)	Injury Description:<br>Extra-articular fractures of the distal tibia<br>Fixation:<br>Ilizarov fixation versus plate osteosynthesis<br>Age: 32.6 years (plate osteosynthesis group) and 32.8 years (Ilizarov fixation group)	40<br>(16F, 26M)	Plate osteosynthesis group: NWB with immobilization initially with progression to partial WBing upon clinical and radiological exam.<br>Ilizarov fixation group: Wbing as tolerated starting post-operative day 1.	Mazur Ankle Score: Ilizarov fixation group, 10 excellent, 10 good; Plate osteosynthesis group, 2 excellent, 8 good, 4 fair, 6 poor.	Two hardware failures in plate osteosynthesis group. None in Ilizarov fixation group (Statistically different between groups).	Plate osteosynthesis group average 196.5 days. Ilizarov fixation group average 130 days. (Statistically different between groups).
Franco-de-la-Torre et al<br>Mexico<br>(tibial shaft)	Injury Description:<br>Tibial shaft fractures<br>Fixation:<br>Intramedullary nail<br>Age: 23.2 years (experimental group) and 26.5 years (control group).	12 (6 per group)<br>(2F, 10M)	Walk twice/day for 20 minutes with walker or crutches; 15% Wbing month 1, 15% month 2, Wbing as tolerated in month 3. Experimental group received noise stimulation on skin surface.	--	--	--

Gross et al<br>United States of America<br>(tibial shaft)	Injury Description:<br>Tibial shaft fractures<br>Fixation:<br>Intramedullary nail<br>Age:<br>WBAT group: 41.8 years.<br>NWB group: 36.1 years	86 patients with 90 fractures<br>(WBAT: 9F)<br>(NWB: 9F)	Immediate WBing as tolerated (50 fractures) vs NWB x 6 weeks (40 fractures).	Short Musculoskeletal Function Assessment (SFMA) administered post op each visit (first 2 weeks). SMFA averages at 6 weeks: Function Wbing as tolerated 35.9, NWB 35.3 (p= 0.90); Bothersome Wbing as tolerated 33.9 NWB 36.6 (p=0.63); Daily activity Wbing as tolerated 54.8, NWB 54.4 (p=0.96); Emotion Wbing as tolerated 29.7, NWB 35.1 (p=0.32), Arm and Hand Wbing Aas tolerated 5.4, NWB 5.1 (p=0.89); Mobility Wbing as tolerated 44.2, NWB 41.0 (p=0.55). SMFA averages at union: Function Wbing as tolerated 24.4, NWB 24.4 (p=0.98); Bothersome Wbing as tolerated 23.7 NWB 24.6 (p=0.90); Daily activity Wbing as tolerated 31.7, NWB 30.9 (0.91); Emotion Wbing as tolerated 27.5, NWB 31.9 (0.43), Arm and Hand Wbing as tolerated 2.5, NWB 2.1 (p=0.74); Mobility Wbing as tolerated 32.4, NWB 30.9 (p=0.78).	Four non-unions: NWB 3, Wbing as tolerated 1 (p=.34). No implant failures. Three delayed unions: NWB 2, Wbing as tolerated 1.	No difference in time to union (Wbing average 22.1 weeks; NWB average 21.3 weeks; p= .76).
Li et al<br>China<br>(calcaneus)	Injury Description:<br>Sanders IV calcaneal fractures<br>Fixation: ORIF<br>Age:<br>Observation group: 39.18 years<br>Control group: 38.75 years	84 (77M, 39F)	Control group: Range of motion exercises and isometrics while NWB initially. Partial Wbing between 13-24 weeks with progressive increase to full Wbing.<br>Observation group: Same exercises but started partial Wbing (25% bodyweight) at four weeks. This increased progressively. Progressed to full Wbing by post-operative week 13.	Maryland Foot Function Scores and American Orthopedic Foot and Ankle Scores (AOFAS) compared before surgery and at weeks 6, 12, and 24 weeks post-operative. Both were significantly higher in the observation group as compared at 6, 12, and 24 weeks post-operative (< 0.001).	No significant difference in angles of Böhler or Gissane between the two groups post-intervention.	--
Sanders et al<br>Canada<br>(lateral malleolus)	Injury Description:<br><br>Weber B closed isolated fractures of lateral malleolus<br><br>Fixation: ORIF versus plaster or fiberglass cast or brace<br><br>Age: 41 years	81 (40F, 41M)	Operative group: NWB 2 weeks, then early mobilization with protected WBing removable cast 3-6 weeks, patient tolerance.<br><br>Non-operative group: Protected Wbing 6 weeks with cast/splint. Both groups trained on axillary crutch use by a physical therapist. Cast and brace discontinued in both after 6 weeks. Full Wbing encouraged for both at this time.	Short Form 36 (SF-36) Physical Component Summary (PCS), 12-weeks operative group 66.6 +/- 18.4 versus non-operative group 60.0 +/- 19.0; joint-specific Olerud-Molander assessment (OMA) of ankle function, 12-weeks op group 61.4 +/- 23.4 vs. non-operative group 56.8 +/- 23.8; (12-weeks had the greatest differences here, but they were not statistically significant. All assessed at first clinic visit, then at 6, 12, 24, and 52 weeks.	Eight in non-operative versus 0 in op group (delayed/non-union). One op versus 8 non-operative (misalignment).	--

[a] RCTs = randomized controlled trials; ATFL = anterior talofibular ligament; ORIF = open reduction internal fixation; F = female; M = male; WBing = weight bearing; NWB = non-weight bearing
