# Supplementary material for: The Safety and Effectiveness of Early, Progressive Weight Bearing and Implant Choice after Traumatic Lower Extremity Fracture: A Systematic Review
Source: Bioengineering (Basel). 2022 Dec 1;9(12):750. doi: 10.3390/bioengineering9120750 (PMC9774827; doi:10.3390/bioengineering9120750)
Supplement: Supplementary file 1 [file bioengineering-09-00750-s001.zip › Table S3_Study Characteristics and Primary Outcomes of Included Non-RCT Studies.pdf]

Table S3. Study Characteristics and Primary Outcomes of Included Non-RCT Studies[a]

Study<br>Country<br>(Bone or joint)	Participants	N	Weight bearing timeline/precautions	Standardized outcome measure scores	Non-union/mal-union rates	Healing time
Braun et al<br>Germany<br>(proximal femur)	Injury Description:<br>Intertrochanteric femur fractures in participants 65 or older<br>Fixation:<br>Cephalomedullary nail osteosynthesis<br>Age: 81.6 years (65-98)	22<br>(16F, 6M)	OpenGo pedobarography insole; daily gait and max/average amount of WBing recorded; physical therapy 30 minutes/day; immediate full WBing allowed with assistive device and WBing as tolerated with activity; average max WBing of 124.7% bodyweight observed.	--	--	--
Cunningham et al<br>United Kingdom<br>(tibia)	Injury Description:<br>Tibia fractures<br>Fixation:<br>External fixation<br>Age: Not reported	27	WBing as tolerated with measurement of force every two weeks.	--	--	--
Ehlinger et al<br>France<br>(peri-prosthetic femur)	Injury Description:<br>12 prosthetic hip fractures, 1 between hip prosthesis and knee, 4 with knee prosthesis<br>Fixation:<br>Locking compression plate<br>Age: 76.7 years	17<br>(15F, 2M)	Immediate full WBing on 10 cases, with partial Wbing (20 kg) 3 cases, NWB 6 weeks 4 cases.	--	--	--

Eid and Deif<br>Egypt<br>(femur)	Injury Description:<br>Group A: Single fracture of femoral shaft<br>Group B: Double fractures of femoral shafts<br>Fixation:<br>Group A: Kuntschner clover-leaf IMN<br>Group B: IMN x 2 (N=2); IMN + ORIF (N=1)<br>Age:<br>Group A: 10-71 years, majority 20-50 years<br>Group B: 30s-40s	106<br>Group A: 103 (37F, 66M)<br>Group B: 3 (1F, 2M)	Both groups used axillary crutches for NWB after 2-3 weeks. WBing determined by radiographic exam. Some with straight cane with questionable callus. Full WBing with bridged callus.	--	14 non-unions out of 103 operations, 11 M/3 F. Increased non-union with earlier WBing (4-6 weeks statistically higher than those after). Group B: 0/3 middle fractures united, others did in 6-8 weeks.	--
Eingartner et al<br>Germany<br>(peri-prostetic)	Injury Description:<br>Periprosthetic fractures of the femur<br>Fixation: IMN<br>Age: 71.2 years (43-86 years)	21<br>(13F, 8M)	Bed rest 1 week-10 days; partial WBing (20 kg) at 14th day; increased Wbing 10 kg/week starting week 12. Reduction in use of axillary crutches at 6 months.	Harris Hip Score, average = 70.5 points (29-95). Follow-up at average of 53.9 months (15-140)	1 delayed/non-union resulting in bone grafting	--
Kim et al<br>South Korea<br>(tibia, fibula)	Patient 1: 47 y/o M, tibial pilon fracture with ORIF.<br>Patient 2: 79 y/o M, tibia-fibula fracture with IMN & ORIF.<br>Patient 3: 33 y/o M, tibial plateau fracture with ORIF	3	3 weeks NWB, Week 1-4 training twice/day, specific percent bodyweight unique to each patient, with body weight support using a lower-body positive-pressure treadmill; Week 1 20-40% bodyweight, gradually increased 10-20%	10-meter Walking Test post training & 6-month follow-up 15.96 & 9.34 (patient 1), 19.73 & 11.93 (patient 2), 26.96 & 8.77 (patient 3).<br>Timed Up-and-Go Test post training & 6-month follow-up 17.15 & 11.2 (patient 1), 18.67 & 12.37 (patient 2), 25.57 & 8.54 (patient 3).<br>Berg Balance Scale pre, post, and 6-month follow-up for patient 1, 37, 41, & 52. Post and 6-month follow-up 36 & 44 for patient 2. Six-month follow-up for patient 3 was 48.	0	--

Martinez et al<br>United States of America<br>(proximal tibia)	Injury Description:<br>Closed proximal tibia fractures<br>Fixation:<br>Fracture bracing<br>Age: 36.3 years	108<br>(19F, 89M)	Progressive WBing as tolerated after discharge with above-the-knee splint. 1 week out, prefabricated functional brace if swelling and acute pain gone. Postponed another week if not (range 4 days to 19.1 weeks, mean 11 days). Progressive WBing as tolerated with axillary crutches. 1 week follow-up post splint application.	--	12% healed with >6 degrees of frontal plane angulation. 2.8% non-union rate. 8 pts (8.4%) had > 10 mm shortening.	Average 17.1 weeks (range 6.6-40.5 weeks)
Millar et al<br>Australia<br>(tibial plateau)	Injury Description:<br>Tibial plateau fractures<br>Fixation:<br>ORIF<br>Age: 50.9 years +/- 13.1	20<br>(11F, 9M)	WBing as tolerated immediately post-operative with axillary crutches as needed for 6 weeks.	Knee Injury and Osteoarthritis Outcome Score (KOOS) - no sport/recreation categories completed. Significant improvements between 3 and 6 months across pain, symptoms, and activities of daily living. Six months to 2 years saw improvement in the symptoms section. Significant difference at each domain at 2 and 6 months when compared to 2 years. Gait speed significantly improved at all timepoints except 1 & 2 years.	--	--
Ohsawa et al<br>Japan<br>(proximal femur)	Injury Description:<br>Intracapsular femur fractures<br>Fixation:<br>Non-operative: assertive early WBing versus conventional rehabilitation<br>Age: Range 79-92 years<br>AG: 86.7 years<br>CG: 87.9 years	20<br>13 AG (12F, 1M)<br>7 CG (6F, 1M)	WBing as tolerated within the first month after beginning of rehab (once patient could sit in wheelchair for endurance purposes).	Pre-hip Function:<br>Face pain scale- AG 0, CG 0 (p = 1)<br>ROM- AG 4, CG 3.7 +/-0.5 (p = 0.373)<br>Ambulation FIM- AG 5, CG 5 (p = 1)<br><br>Post-hip Function:<br>Face pain scale- AG 12.6 +/-4.8, CG 10 (p = 0.390)<br>ROM- AG 4.1+/-0.3, CG 3.9+/-0.7 (p = 0.324)<br>Ambulation FIM- AG 18.2+/-7.9, CG 9.4+/-4.3 (p = 0.00135)	--	--

Ostermann et al<br>Germany<br>(distal femur)	Injury Description:<br>Unicondylar femur fractures<br>Fixation:<br>Open anatomic reduction, temporary fixation with Kirschner wires; definitive fixation with cancellous screws<br>Age: 33 years, range from 13-73 years	24<br>(5F, 19M)	1 week out allowed to "eggshell" weight bear. Full WBing allowed after 6-8 weeks if no concomitant injuries.	Neer score: 20 excellent (average 91 units), 3 satisfactory (average 77 units), 1 unsatisfactory.	--	--
Schildhauer et al<br>Germany<br>(calcaneal fracture)	Injury Description:<br>Depression-type calcaneal fractures<br>Fixation:<br>ORIF augmented with calcium phosphate cement<br>Age: 22-65 years	32<br>(32M)<br>with 36 fractures	Full WBing 8-12 weeks vs full WBing 3-6 weeks but only progressed to full WBing if first few patients were able to perform full WBing at 12 weeks successfully (i.e., the first 3 patients were not allowed to fully weight bear until 12 weeks. After achieving good clinical results, patients 4-16 were allowed to transition to full WBing at 8 wks. Last 3 patients were full WBing at 3 weeks).	Kerr Calcaneal Score (Pain 36 points, Work 25 points, Walking 25 points, Walking aid 14 points)<br>Average Kerr Calcaneal Score: 80.2 (Range 48-100). WBing 8 or 12 weeks post op averaged score 81.4; WB 3, 4 or 6 weeks post operative average score 78.9 (p = 0.56).	No loss of reduction at 3 weeks seen.	--
Thewlis et al<br>Australia<br>(tibial plateau)	Injury Description:<br>Partial articular tibial plateau fractures<br>Fixation:<br>ORIF<br>Age: 51.4 years.	9<br>(4F, 5M)	WBing as tolerated immediately post-operative with assistive devices for balance and comfort.	Gait speed: Significant improvement over time, 2, 12, 26, and 52 weeks, with the mean differences at 2-26, 2-52, and 12-52.	--	--
Zhang et al<br>China<br>(calcaneal fractures)	Injury Description:<br>Intra-articular calcaneal fractures<br>Fixation:<br>Reduction with external fixation in calcaneus fixation boot<br>Age: 35 years with range from 18-37	102<br>(27F, 75M)<br>with 107 fractures	Initiated WBing functional exercise in boot within 24 hours, increased after 2 weeks, 3-4 weeks with crutches, WBing without assistive device in 8-10 weeks.	Reported outcomes: 53 excellent, 41 good, 9 fair, 4 poor.	--	--

[a] Non-RCT = non-randomized controlled trial; F = female; M = male; WBing = weight bearing; NWB = non-weight bearing; IMN = intramedullary nail; ORIF = open reduction internal fixation; AG = assertive group; CG = conventional group.
